# Molecular basis for protection of ribosomal protein L4 from cellular degradation

**DOI:** 10.1038/ncomms14354

**Published:** 2017-02-02

**Authors:** Ferdinand M. Huber, André Hoelz

**Affiliations:** 1 Division of Chemistry and Chemical Engineering, California Institute of Technology, 1200 East California Boulevard, Pasadena, California 91125, USA

## Abstract

Eukaryotic ribosome biogenesis requires the nuclear import of ∼80 nascent ribosomal proteins and the elimination of excess amounts by the cellular degradation machinery. Assembly chaperones recognize nascent unassembled ribosomal proteins and transport them together with karyopherins to their nuclear destination. We report the crystal structure of ribosomal protein L4 (RpL4) bound to its dedicated assembly chaperone of L4 (Acl4), revealing extensive interactions sequestering 70 exposed residues of the extended RpL4 loop. The observed molecular recognition fundamentally differs from canonical promiscuous chaperone–substrate interactions. We demonstrate that the eukaryote-specific RpL4 extension harbours overlapping binding sites for Acl4 and the nuclear transport factor Kap104, facilitating its continuous protection from the cellular degradation machinery. Thus, Acl4 serves a dual function to facilitate nuclear import and simultaneously protect unassembled RpL4 from the cellular degradation machinery.

## Abstract (web summary)

Acl4 is a dedicated assembly chaperone for ribosomal protein RpL4 that recognizes RpL4 in the cytoplasm to facilitate its nuclear import. Here the authors reveal the mechanism whereby Acl4 recognizes RpL4 and functions to protect it from Tom1-mediated degradation until RpL4 incorporation into the maturing 60S pre-ribosomal subunit.

The spatial separation of cytoplasmic protein translation and nucleolar ribosome biogenesis requires the nuclear import of ∼80 nascent ribosomal proteins (RPs) through the nuclear pore complex (NPC) and subsequent export of pre-ribosomal subunits into the cytoplasm^1^,^2^. These NPC-dependent nucleocytoplasmic transport events generate a layer of regulation that facilitates the dynamic adjustment of total ribosome numbers along with RP quality control and rapid degradation^3^,^4^,^5^,^6^,^7^. Ribosome biogenesis is temporally and spatially coordinated by ∼200 *trans*-acting ribosome assembly factors that mediate the hierarchical assembly of pre-ribosomal subunits^8^. In addition, dedicated assembly chaperones assist ribosome biogenesis by recognizing and facilitating transport of nascent RPs to the pre-ribosome^9^,^10^,^11^,^12^,^13^,^14^,^15^,^16^.

In the mature ribosome, ribosomal proteins form multiple intricate interactions with both neighbouring RPs and ribosomal RNA (rRNA)^17^. Contacts with rRNA are mediated predominantly by electrostatic contacts between the phosphate backbone and arginine- and lysine-enriched motifs of RP elements located at their termini or within protruding loops^17^. The elongated ∼70-residue loop of ribosomal protein L4 (RpL4) is devoid of secondary structure elements and extends deep into the large ribosomal subunit core, forming a series of interactions with rRNA and lines the peptide exit tunnel^17^,^18^. The mechanism by which nascent ribosomal proteins escape unfavourable interactions with nucleic acids, other RPs, and the cellular degradation machinery remains poorly understood. We have previously shown that the dedicated assembly chaperone Acl4 recognizes nascent RpL4, facilitates its nuclear import, and releases RpL4 on engaging RpL18 at the pre-ribosome^10^. Moreover, Acl4 is required for the soluble expression of newly synthesized RpL4 and for the protection of RpL4 from the Tom1-dependent cellular degradation machinery^16^,^19^. Therefore, we hypothesized that Acl4 could generate a protective environment for RpL4 by sequestering elongated RpL4 elements until its incorporation into the pre-ribosome.

Here, we report the 2.4-Å resolution crystal structure of RpL4 in complex with its dedicated ribosome assembly chaperone Acl4. The structure reveals an extensive interaction encompassing 70 exposed residues of the internal RpL4 loop that are sequestered by the concave Acl4 surface on complex formation. The observed binding mode differs dramatically from canonical chaperone–substrate interactions that generally recognize short exposed hydrophobic peptide stretches. Despite the considerable binding interface in the Acl4·RpL4 complex, we identified a single Acl4 residue that abolishes RpL4 binding and may serve as an intrinsic weak spot for complex disassembly. Moreover, we determined the 3.0-Å resolution crystal structure of the karyopherin transport factor Kap104 in complex with the eukaryote specific RpL4 extension. Our structural and biochemical analysis demonstrates that the RpL4 extension possesses overlapping binding sites for a second Acl4 copy and Kap104. Whereas, unprotected RpL4 is targeted by the E3-ubiquitin ligase Tom1 for proteasome-dependent degradation, interactions with Acl4 and Kap104 sequester Tom1 ubiquitination sites in the RpL4 loop and extension. Thus, ribosome assembly chaperones can serve dual functions by facilitating nuclear import while simultaneously protecting unassembled ribosomal proteins from the cellular degradation machinery.

## Results

### Crystal structure of the Acl4·RpL4 complex

**Figure 1:**
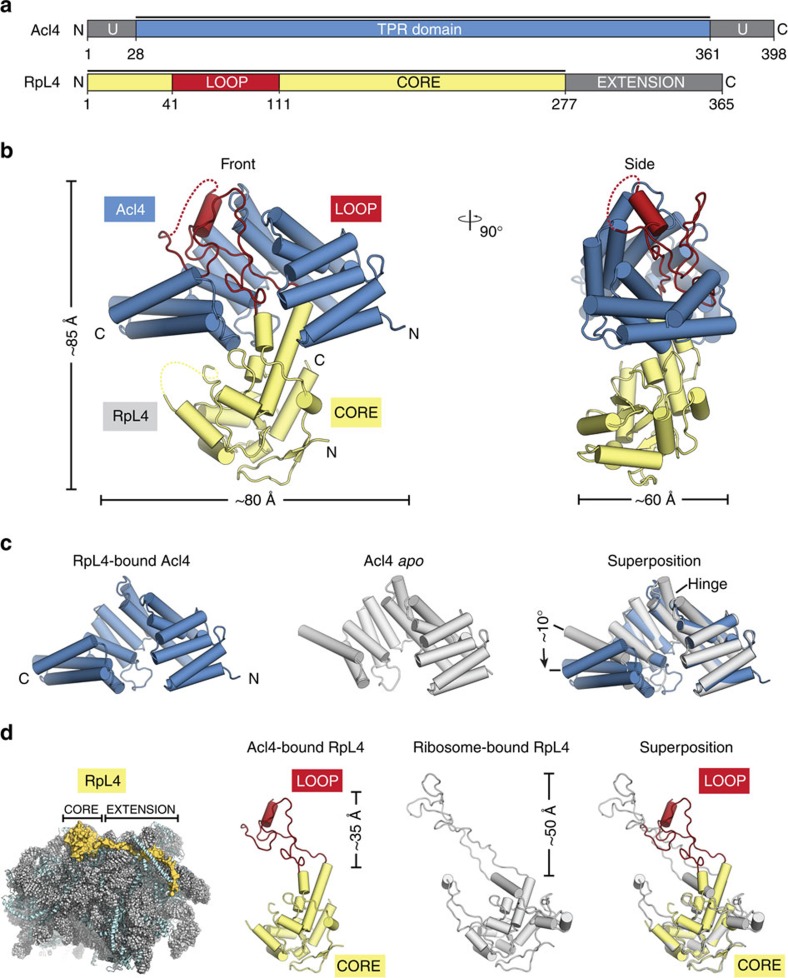
Analysis of the Acl4·RpL4 structure. (**a**) Domain representation of Acl4 and RpL4 from *Chaetomium thermophilum*. Acl4: unstructured N- and C-terminal regions (dark grey); central TPR domain (blue). RpL4: core (yellow); loop (red); C-terminal extension (dark grey). Black bars represent crystallized fragments. (**b**) Crystal structure of the *Chaetomium thermophilum* Acl4·RpL4 complex, shown in cartoon representation. A 90° rotation is shown on the right. Colouring is according to panel **a**. (**c**) Superposition of RpL4-bound Acl4 (blue) with Acl4 *apo* (grey) (PDB ID 4YNV)^10^. (**d**) Cartoon representation of the *S. cerevisiae* large ribosomal subunit (PDB ID 4V88) showing RNA (grey), proteins (teal), and RpL4 (yellow)^17^. Superposition of Acl4-bound RpL4 with ribosome-bound RpL4 (grey).

We set out to identify the molecular basis of Acl4-dependent RpL4 sequestration and protection. We generated a biochemical interaction map between RpL4, its assembly chaperone Acl4, and its transport factor Kap104 and gained insight into the Acl4–RpL4 binding mode at the atomic level. Crystals of the *Chaetomium thermophilum* Acl4·RpL4 complex, which included the Acl4 TPR domain (residues 28 to 361) and the globular core of RpL4 (RpL4^CORE^) and the entire elongated loop (RpL4^LOOP^), diffracted to 2.4-Å resolution (Fig. 1a). The structure was solved by single-wavelength anomalous dispersion (SAD) using Seleno-L-methionine (SeMet) labelled proteins. The final model was refined to R_work_ and R_free_ values of 19.1% and 22.7%, respectively, with excellent stereochemistry (Table 1).

Acl4 adopts an α-helical tetratricopeptide repeat (TPR) fold composed of seven TPRs (αA-αN) and a C-terminal flanking helix (αO) with an overall right-handed superhelical twist that accommodates the entire 70-residue RpL4^LOOP^ with its concave surface (Fig. 1b, Supplementary Fig. 1 and Supplementary Movie 1). Whereas, RpL4^LOOP^ forms numerous contacts with the Acl4 surface, RpL4^CORE^ contributes few additional interactions to the Acl4·RpL4 complex. Comparing the Acl4·RpL4 structure to our previously determined Acl4 *apo* structure revealed a conformational change on RpL4^LOOP^ binding, which is unusual for TPR domains (Fig. 1c)^10^. The longer central Acl4 helices αF and αG form a hinge between the N-terminal (αA-αF) and C-terminal halves (αG-αO) of Acl4, which rotate as rigid bodies by ∼10° from an open to a closed conformation on binding RpL4 (Fig. 1c).

### RpL4^LOOP^ undergoes dramatic rearrangement on Acl4 binding

In the mature ribosome, the RpL4^LOOP^ adopts a remarkably elongated conformation, reaching deep into the rRNA core of the large ribosomal subunit, while the ∼100-residue RpL4 extension, RpL4^EXT^, extends ∼120 Å over the ribosomal surface (Fig. 1d)^17^. Whereas, the conformation of RpL4^CORE^ remains largely unchanged, comparison of Acl4- and ribosome-bound RpL4 revealed a striking conformational change of the elongated RpL4^LOOP^ (Fig. 1d). Within the ribosome, RpL4^LOOP^ is fully extended and reaches ∼50 Å into the centre of the large subunit^17^. In contrast, binding to Acl4 results in a great compaction of RpL4^LOOP^ by more than ∼15 Å, sequestering a maximum number of residues into the protective environment of the concave Acl4 surface (Fig. 1d). Acl4-binding induces the formation of an α-helix within RpL4^LOOP^ (α3, residues 89 to 97), which is entirely devoid of secondary structure elements in the context of the intact ribosome. Thus, both Acl4 and RpL4 undergo dramatic conformational changes on complex formation.

**Figure 2:**
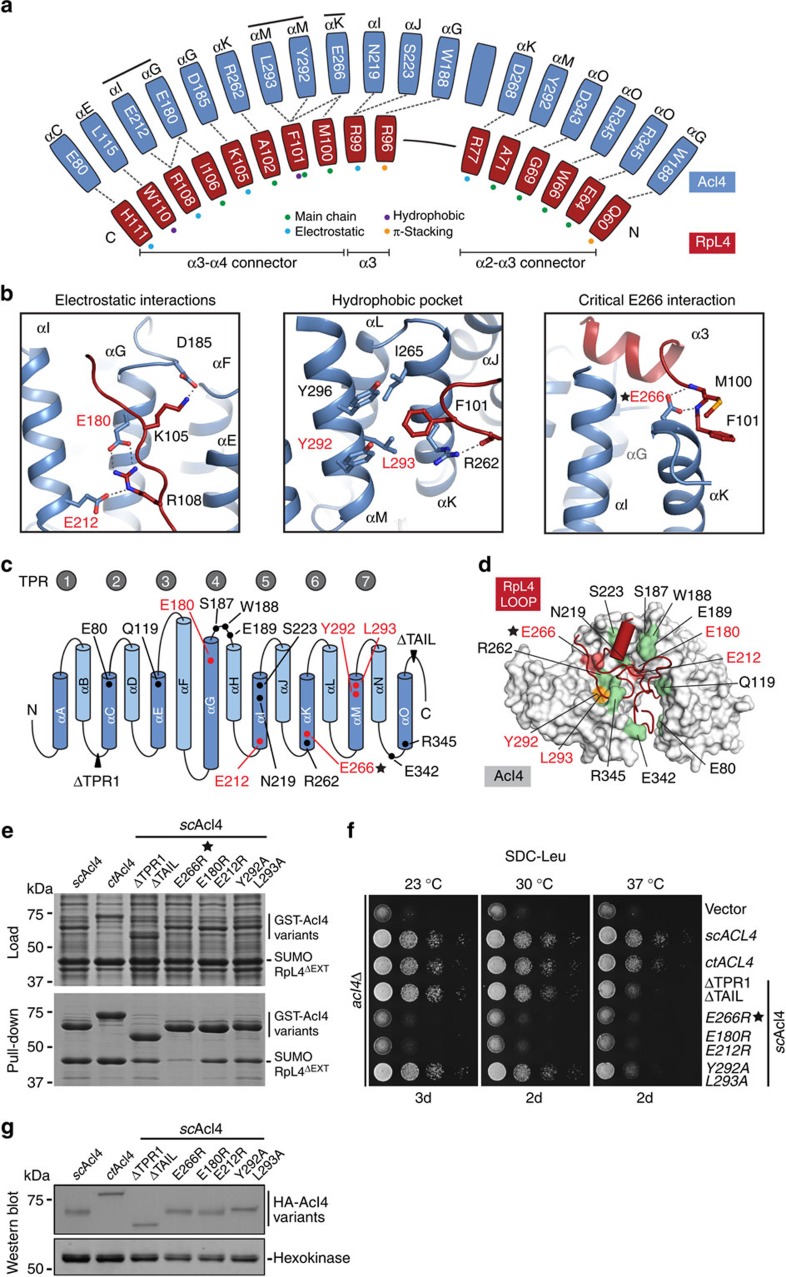
Acl4·RpL4 interaction analysis. (**a**) Schematic representation of the Acl4–RpL4 interface, coloured as in Fig. 1. Coloured dots indicate the interaction type between depicted residues. (**b**) Acl4·RpL4 interaction hotspots. Boxes show three interactions between RpL4 (red) and Acl4 (blue) in cartoon representation. (**c**) Schematic representation of the Acl4 TPR domain fold architecture. The positioning of RpL4 interaction residues is indicated. (**d**) Mutational analysis of the Acl4–RpL4 interaction. Acl4 (grey) and RpL4^LOOP^ (red) are shown in surface and cartoon representation, respectively. Mutated Acl4 residues are plotted on the surface and coloured according to effect on RpL4 binding: green, no effect; orange, medium effect; and red, strong effect. (**e**) Interaction analysis of Acl4 and RpL4^ΔEXT^. Pull-down interaction analysis between *S. cerevisiae* GST-Acl4 variants (bait) and RpL4^ΔEXT^. Loaded (top) and pulled-down (bottom) fractions are indicated and Acl4 mutations are depicted above each lane. (**f**) Growth analysis of Acl4 variants. Residue numbering is according to *C. thermophilum* Acl4. (**g**) Western blot analysis of the expression levels of Acl4 variants in *S. cerevisiae*. HA-tagged Acl4 variants and the hexokinase loading control were detected with anti-HA and anti-hexokinase antibodies, respectively.

The majority of RpL4^LOOP^ is buried by the concave Acl4 surface and involves several interactions formed by predominantly invariant Acl4 residues (Supplementary Figs 2–4). The extensive nature of the interactions is best illustrated by the sheer number of residues directly involved in Acl4–RpL4^LOOP^ binding: 42 out of 70 RpL4^LOOP^ residues and 87 out of 333 Acl4 residues (Fig. 2a; Supplementary Fig. 2). The interface is formed primarily by electrostatic interactions between the acidic Acl4 surface and the basic RpL4^LOOP^ (Supplementary Fig. 4b,d). However, additional hydrophobic and π-stacking interactions contribute to the stability of the Acl4·RpL4 complex as well (Fig. 2a,b).

### Acl4·RpL4 harbours an intrinsic weak spot for disassembly

Although, the extensive Acl4·RpL4 interface formed by a considerable number of direct interactions is ideally suited for substrate protection, this simultaneously represents a challenge for the eventual dismantling of the complex during ribosome biogenesis. To identify the underlying molecular mechanism, we employed a comprehensive structure- and conservation-guided mutagenesis approach with the goal of identifying Acl4 residues capable of triggering the disassembly of the Acl4–RpL4 interaction (Fig. 2a–e). Individual mutations of most of the invariant Acl4 residues proved to be insufficient to disrupt or even weaken the Acl4–RpL4 interaction (Supplementary Fig. 5). We next focused on two highly conserved interaction sites in the concave Acl4 surface: the electrostatic interactions of Acl4 residues Glu180 and Glu212, both of which form a salt-bridge with RpL4 Arg108, and a hydrophobic pocket formed by Acl4 residues Tyr292 and Leu293, which engage RpL4 Phe101 (Fig. 2b). However, neither the Acl4 E180R/E212R charge-swap nor the Acl4 Y292A/L293A double mutation had a major effect on the interaction with RpL4 (Fig. 2b–d; Supplementary Fig. 5). In contrast, we identified a single charge-swap Acl4 mutation, E266R that abolished the Acl4–RpL4^LOOP^ interaction almost completely (Supplementary Fig. 5). Glu266 is located on the top surface of Acl4·RpL4 and forms hydrogen bonds with the mainchain amides of RpL4 residues Met100 and Phe101, thereby anchoring the C-terminal end of RpL4 helix α3 and compressing the RpL4^LOOP^ to the Acl4 surface (Fig. 2b). Introducing the identified Acl4 mutants of the crystallized *Chaetomium thermophilum* protein into its *Saccharomyces cerevisiae* homologue established that the overall architecture of the Acl4·RpL4 complex is evolutionarily conserved (Fig. 2e; Supplementary Fig. 5). In fact, despite limited sequence conservation, *C. thermophilum* Acl4 is capable of forming a chimeric complex with *S. cerevisiae* RpL4 (Fig. 2e). These results suggest that the Glu266-mediated interactions constitute an intrinsic weak spot that is critical for Acl4·RpL4 complex disassembly. A structural comparison with the *apo* Acl4 reveals that disrupting these interactions upon engaging the pre-ribosomal surface leads to the simultaneous relaxation of the Acl4 TPR domain and elongation of RpL4^LOOP^, reminiscent of a spring-loaded mechanism (Supplementary Movie 2).

To validate the physiological relevance of our findings on the mechanism of Acl4–RpL4 binding and disassembly, we generated an *S. cerevisiae* Acl4 deletion (*acl4Δ*) strain and analysed various Acl4 mutants. Deletion of TPR1 and the acidic C-terminal region of Acl4 caused a growth defect at 37 °C, as did mutation of the conserved hydrophobic pocket with Tyr292 and Leu293 to alanines, consistent with the biochemical findings. In contrast, the E266R mutant displayed a severe growth defect, identical to the *acl4Δ* phenotype, demonstrating the physiological relevance of closely anchoring RpL4^LOOP^ to the Acl4 surface. Notably, the Acl4 E266R mutation exhibited identical behaviour in size exclusion chromatography, confirming that the observed effect was not caused by improper Acl4 E266R protein folding (Supplementary Fig. 6). Surprisingly, the E180R/E212R double charge-swap mutation of the electrostatic binding site, which only moderately affected Acl4–RpL4 binding, also caused a substantial growth defect at all analysed temperatures, suggesting a role of this binding site for the proper release of RpL4 into the maturing ribosome. Notably, all Acl4 variants were expressed at similar levels and predominantly localized to the nucleus with the RpL4-binding deficient mutants only displaying a slight increase in cytoplasmic localization (Fig. 2g; Supplementary Fig. 7).

### Acl4 and Kap104 share an overlapping binding site on RpL4^EXT^

**Figure 3:**
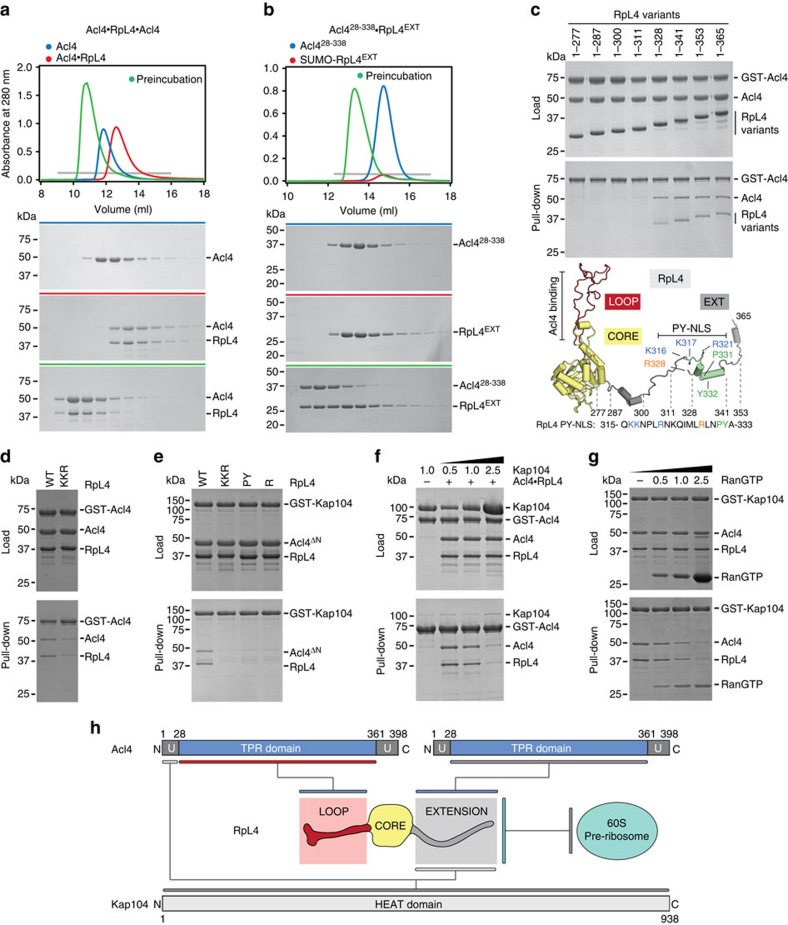
Biochemical Acl4·RpL4·Kap104 interaction map. (**a**,**b**) Size exclusion chromatography (SEC) analysis of Acl4, Acl4·RpL4, and SUMO-RpL4^EXT^. SEC profiles of proteins or protein complexes are shown individually (blue and red) and after preincubation (green). (**c**) GST pull-down of pre-purified GST-Acl4 and Acl4·RpL4 C-terminal truncation variants. Loaded (top) and pulled-down (bottom) fractions are shown. Cartoon representation of RpL4 with RpL4^LOOP^, RpL4^CORE^, and RpL4^EXT^ coloured as in Fig. 1a. The basic PY-NLS is coloured in green and the analysed fragment boundaries are indicated. As reference, the primary sequence of the basic PY-NLS and the consensus residues are shown. (**d**) GST pull-down with pre-purified GST-Acl4 and Acl4·RpL4 variants. (**e**) GST pull-down with pre-purified GST-Kap104 and Acl4^ΔN^·RpL4 variants. Labelling indicates RpL4 variants (WT, wild type; KKR, K316A/K317A/R321A; PY, P331A/Y332A; R, R328A). (**f**) GST pull-down with pre-assembled Acl4·RpL4·GST-Acl4 and increasing amounts of Kap104. (**g**) GST pull-down with pre-assembled Acl4·RpL4·GST-Kap104 and increasing amounts of RanGTP. (**h**) Schematic representation of the Acl4·RpL4·Kap104 interaction map.

By employing a more sensitive size exclusion chromatography assay, we identified an additional interaction between Acl4 and RpL4^EXT^, which was previously missed in GST pull-down and yeast two-hybrid assays^10^,^16^. We found that the heterodimeric Acl4·RpL4 complex is capable of interacting with an additional Acl4 molecule resulting in the formation of a heterotrimeric Acl4·RpL4·Acl4 complex with a 2:1 stoichiometry that is evolutionarily conserved (Fig. 3a; Supplementary Fig. 8a). Mapping of the binding site established that RpL4^EXT^ is necessary and sufficient for binding of the second Acl4 copy (Fig. 3b). Further truncation analysis identified an 18-residue region encompassing residues 311–328 of RpL4^EXT^ that is required for Acl4·RpL4·Acl4 complex formation. Alanine substitution of the three highly conserved basic residues in this region, Lys316, Lys317 and Arg321, substantially reduced binding of the second Acl4 copy to Acl4·RpL4 (Fig. 3b–d). Notably, we observed no Acl4 exchange from the RpL4^LOOP^ binding site in the conditions of the performed pull-down experiments, demonstrating that the Acl4·RpL4 heterodimer is very stable in solution.

Previously, we found that the transport factor Kap104 binds to Acl4·RpL4 to form a heterotrimeric complex with equimolar stoichiometry^10^. Further mapping revealed that two distinct regions in Acl4·RpL4 are sufficient for Kap104 binding, one located in RpL4^EXT^ and another in the basic unstructured 28-residue N-terminal region of Acl4 (Fig. 3h; Supplementary Fig. 9). Consistently, the binding between Acl4·RpL4 and Kap104 is abolished when both regions are deleted (Supplementary Fig. 9c). RpL4^EXT^ harbours a canonical basic phenylalanine-tyrosine nuclear localization signal (PY-NLS) and alanine mutagenesis confirmed that all canonical elements of its consensus sequence are critical for the Kap104–RpL4^EXT^ interaction (Fig. 3e)^20^. Because Acl4 and Kap104 bind to overlapping sites in RpL4^EXT^ and Acl4·RpL4 possesses a second Kap104 binding site, we tested whether Kap104 is able to displace the RpL4^EXT^-bound Acl4 copy. Indeed, in a competition experiment Kap104 replaced the RpL4^EXT^-bound Acl4 copy to form an Acl4·RpL4·Kap104 complex (Fig. 3f). As expected, RanGTP released Acl4·RpL4 from this nuclear import heterotrimer (Fig. 3g). In addition, the nuclear import adaptor Kap-α was also able to form a heterotrimeric Acl4·RpL4·Kap-α nuclear import complex, indicating that multiple karyopherins are capable of transporting Acl4·RpL4 to the nucleus (Supplementary Figs 8d,9). However, Kap104 displaced Kap-α from the Acl4·RpL4·Kap-α heterotrimer in direct competition experiments, suggesting that Kap104 is the primary nuclear import factor for Acl4·RpL4 (Supplementary Fig. 8e).

### Acl4 and Kap104 protect nascent RpL4 from degradation

**Figure 4:**
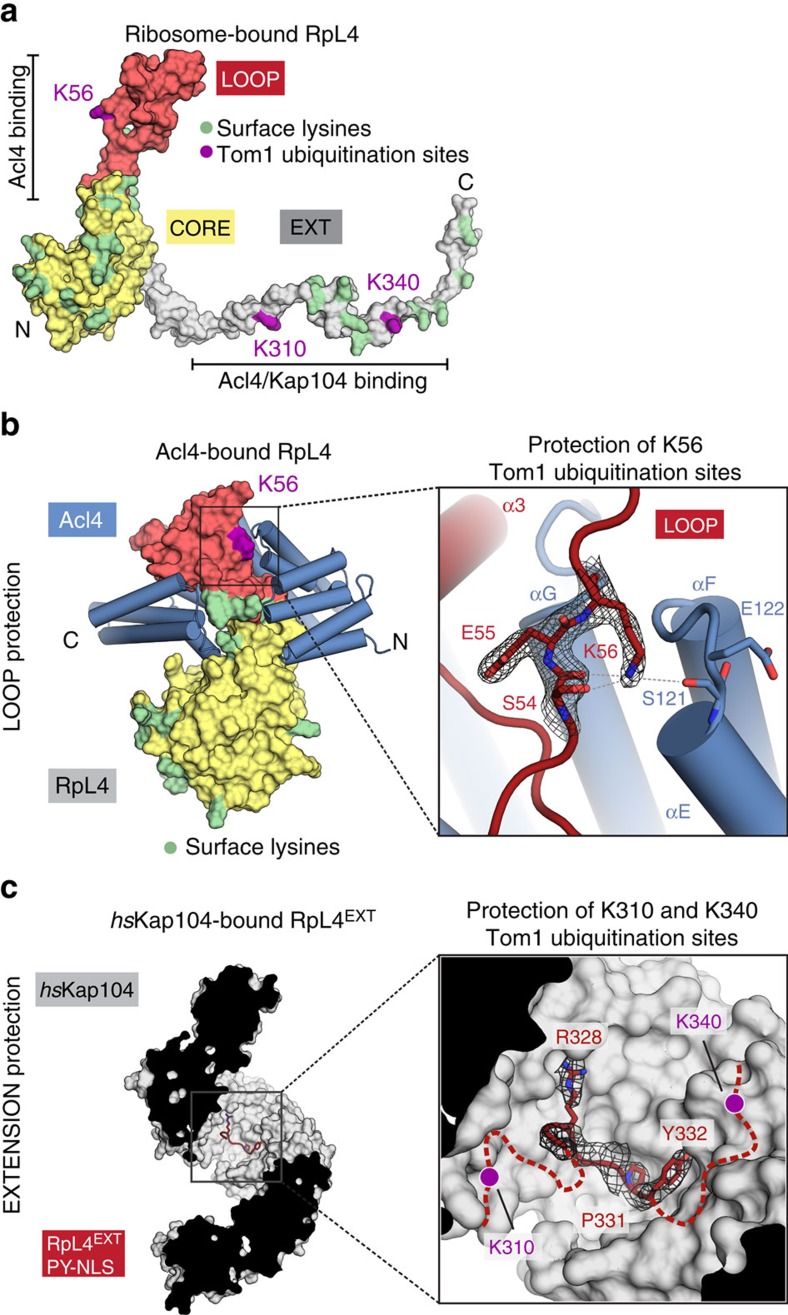
Shielding of Tom1 ubiquitination sites. (**a**) Surface representation of ribosome-bound RpL4 (PDB ID 4V88)^17^. Acl4 and Kap104 binding sites are indicated with black bars. (**b**) Acl4-bound RpL4 (coloured as in Fig. 1a) and Acl4 (blue) are shown in surface and cartoon representation, respectively. The inset marks the Tom1 ubiquitination site that is illustrated in detail on the right. Acl4 (blue) and RpL4 (red) and critical residues highlighted in stick representation with a section of the final 2|F_o_|-|F_c_| electron density map contoured at 1.0 σ. (**c**) Crystal structure of the *hs*Kap104·RpL4^EXT^ complex. The inset marks the location of the Kap104 PY-NLS binding site that is illustrated in detail on the right. The residues of the PY-NLS consensus sequence, Arg328, Pro331 and Tyr332 are highlighted in stick representation with a section of the final 2|F_o_|-|F_c_| electron density map contoured at 1.0 σ. Magenta circles indicate the approximate location of RpL4 residues K310 and K340 that are ubiquitinated by Tom1 in the absence of Kap104.

We previously described a novel pathway for excess ribosomal protein quality control (ERISQ) involving the E3 ubiquitin ligase Tom1, which marks excess ribosomal proteins for proteasome-dependent degradation^19^. RpL4^LOOP^ residue Lys56 along with RpL4^EXT^ residues Lys310 and Lys340 were identified as Tom1 recognition sites, which were ubiquitinated in the absence of Acl4 and Kap104 (Fig. 4a)^19^. The crystal structure of Acl4·RpL4 now shows that Lys56 is located in the highly conserved RpL4^LOOP^ and is sequestered by the Acl4 surface, thus shielded from Tom1-mediated ubiquitination (Fig. 4b). These findings demonstrate that in the RpL4-binding deficient Acl4 E266R mutant Lys56 in RpL4^LOOP^ is not sequestered by Acl4 and therefore is a target for Tom1-dependent ubiquitination. Thus, the growth defect observed in the Acl4 E266R mutant likely is the consequence of Tom1-dependent RpL4 ubiquitination and degradation, resulting in reduced soluble levels of RpL4 and in turn of 60S pre-ribosomal particles^10^,^16^.

To explore whether RpL4^EXT^ residues Lys310 and Lys340 are protected by Kap104 in a similar fashion, we determined the crystal structure of Kap104 in complex with RpL4^EXT^ to 3.0 Å resolution. The Kap104·RpL4^EXT^ structure revealed that the PY-NLS of RpL4^EXT^ engages the concave surface of Kap104 in the same binding mode as previously established for other PY-NLS sequences^20^. Upon Kap104 binding to RpL4^EXT^ and formation of a nuclear import complex both Tom1-modification sites of RpL4^EXT^ are sequestered by the concave Kap104 surface, consistent with our previous protection results of an *in vitro* Tom1 ubiquitination assay (Fig. 4c)^19^.

**Figure 5:**
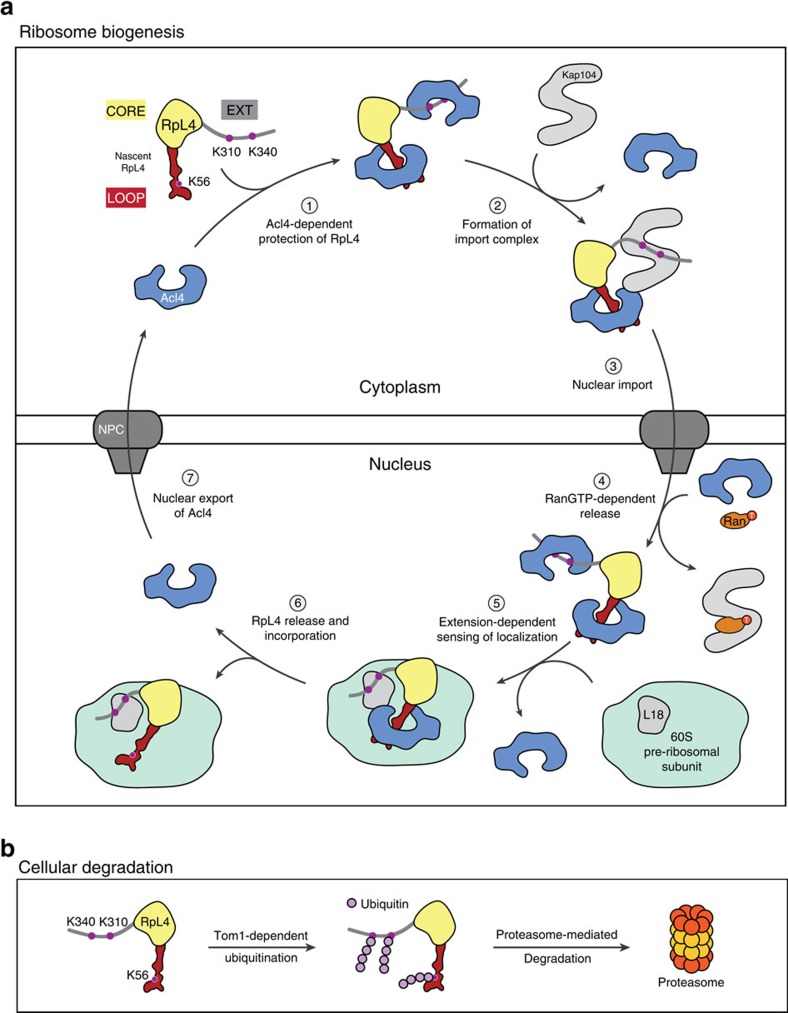
Model for nuclear import and balancing of RpL4. (**a**) Acl4- and Kap104-mediated nuclear import of RpL4. The cycle involves seven steps. (1) Following translation, nascent RpL4 is protected by two copies of Acl4 at its unstructured loop and at the unstructured C-terminal extension. (2) A stoichiometric hetero-trimeric nuclear import complex is formed by binding of Acl4·RpL4 to the transport factor Kap104. Kap104 binding occurs in a bi-partite fashion and involves the basic unstructured N-terminal region of Acl4 and RpL4^EXT^, displacing the RpL4^EXT^-bound Acl4 copy. (3) Kap104 dependent transport of Acl4·RpL4 through the NPC. (4) After successful transport, the Acl4·RpL4·Kap104 import complex is disassembled by nuclear RanGTP, releasing Acl4·RpL4 into the nucleoplasm. (5) RpL4^EXT^ contacts RpL18 and expansion segment 7 on the surface of the pre-60S subunit^10^. (6) Constructive interactions result in disassembly of the Acl4·RpL4 complex and incorporation of RpL4 into the large pre-ribosomal subunit. (7) Potential nuclear export of Acl4 allows its entering into the next RpL4 transport cycle. (**b**) Balancing of excess unassembled ribosomal proteins. In the absence of Acl4 and Kap104, unassembled RpL4 is ubiquitinated by Tom1 and degraded by the proteasome-dependent degradation machinery^19^.

In summary, these results together with our previous findings allow us to propose a model of the entire RpL4 life cycle (Fig. 5): Nascent RpL4 binds two Acl4 copies, one via RpL4^LOOP^ and another via RpL4^EXT^. Kap104 replaces one Acl4 copy and shuttles Acl4·RpL4 across the nuclear envelope. Once in the nucleus, Kap104 releases RpL4^EXT^ on RanGTP binding allowing the rebinding of a second Acl4 copy from the nuclear Acl4 pool. RpL4 release from Acl4 and ribosome incorporation is dependent on the interaction of RpL4^EXT^ with RpL18 and is triggered by relaxation of the presumably spring-loaded Acl4·RpL4 complex at the pre-60S ribosomal subunit (Fig. 5a)^10^. Whereas, unprotected RpL4 is recognized and ubiquitinated by the E3 ligase Tom1, followed by its proteasome-dependent degradation, protection of RpL4 by Acl4 and Kap104 generates a pool of RpL4 available for ribosome biogenesis^16^,^19^. Thus, ribosome assembly chaperones not only facilitate nuclear import and pre-ribosome incorporation of their ribosomal protein substrates, but are also essential for their protection from the cellular degradation machinery (Fig. 5b). It remains an open question how Acl4 and other ribosome assembly chaperones return to the cytoplasm after their substrate RPs are incorporated in the pre-ribosomal particle and whether this occurs in a karyopherin-dependent manner. However, the presence of only sub-stoichiometric amounts of Acl4 in the cell strongly suggests that Acl4 shuttles between nucleus and cytoplasm. Furthermore, because the NPC allows passive diffusion of small proteins with a mass of less than ∼40 kDa, the re-export of free Acl4 may not require a dedicated transport factor. The next important steps will be to identify and characterize the assembly chaperones for the remaining ∼70 ribosomal proteins to establish whether the principles identified for Acl4 are conceptually similar. Additionally, the development of an *in vitro* ribosomal assembly system will be essential for the elucidation of the complex interplay of chaperoned ribosomal proteins, the cellular degradation machinery, and the maturing pre-ribosomal particle.

Unlike promiscuous folding chaperones that recognize exposed short hydrophobic secondary structure elements, Acl4 serves a dedicated sequestering function and harbours an intrinsic trigger for RpL4 release. Thus, the Acl4–RpL4 interaction constitutes a prototype for a dedicated assembly chaperone–substrate interaction that exerts multiple functions. We envision that a similar mechanism is employed by other ribosomal assembly chaperones and by assembly factors of other multi-component macromolecular machineries.

## Methods

### Bacterial and yeast expression constructs

*S. cerevisiae* and *C. thermophilum* DNA fragments of Acl4, RpL4, Kap104 and Kap-α and of *Homo sapiens* Ran were amplified by PCR and ligated into bacterial expression vectors pGEX-6P-1 (GE Healthcare), a modified pET28a and pETDuet1 vector (both Novagene) that contained an N-terminal His_6_-SUMO (small ubiquitin-like modifier) tag (pET28a-SUMO, pETDuet1-SUMO), and a modified pET28a vector (Novagene) containing an N-terminal His_6_ tag followed by a PreScission cleavage site^21^,^22^. The expression construct of *H. sapiens* Kap104 in which the internal loop residues 337–367 were replaced with a GGSGGSG linker was a gift from Yuh Min Chook^20^. Acl4 and RpL4 variants were amplified by PCR and ligated into yeast expression vectors pRS415, pRS415-mCherry, pRS415-HA-mCherry and pRS413-eGFP. Mutants were generated using QuikChange mutagenesis (Stratagene) and confirmed by DNA sequencing. Details of all bacterial and yeast expression constructs are summarized in Supplementary Tables 1 and 2.

### Protein expression and purification

Bacterial expression constructs were transformed in *Escherichia coli* BL21-CodonPlus(DE3)-RIL cells (Stratagene) and grown in LB medium to an OD_600_ of ∼0.6 before induction with 0.5 mM isopropyl β-D-thiogalactoside (IPTG). Cultures containing *C. thermophilum* protein expression constructs were grown for 18 h at 23 °C, while *S. cerevisiae* and *H. sapiens* proteins were expressed for 18 h at 18 °C. Cells were harvested by centrifugation and resuspended in a buffer containing 20 mM TRIS (pH 8.0), 500 mM NaCl, 5 mM β-mercaptoethanol (β-ME), 2 μM bovine lung aprotinin (Sigma) and complete EDTA-free protease inhibitor cocktail (Roche) and flash frozen in liquid nitrogen. Cells were supplemented with 1 mg DNase I (Roche), lysed with a cell disrupter (Avestin) and centrifuged at 4 °C with 40,000*g* for 1 h. The supernatant was filtered through a 0.45 μm filter (Millipore).

#### Purification of His_6_-SUMO-tagged Acl4, RpL4, Acl4·RpL4 and Kap-α variants

Filtered lysate of His_6_-SUMO tagged proteins was applied to a Ni-NTA column (Qiagen) equilibrated with a buffer containing 20 mM TRIS (pH 8.0), 500 mM NaCl and 5 mM β-ME and eluted with a linear imidazole gradient. Protein-containing fractions were pooled and cleaved with ubiquitin-like-specific protease 1 (ULP1) and dialysed against a buffer containing 20 mM TRIS (pH 8.0), 100 mM NaCl and 5 mM β-ME (His_6_-SUMO-RpL4^EXT^ was dialysed but not treated with ULP1). Dialysed proteins were applied to a Ni-NTA column equilibrated with a buffer containing 20 mM TRIS (pH 8.0), 100 mM NaCl and 5 mM β-ME and the unbound fraction was loaded onto a HiTrapQ HP (GE Healthcare) ion exchange column equilibrated in a buffer containing 20 mM TRIS (pH 8.0), 100 mM NaCl, and 5 mM DTT and eluted with a linear salt gradient. Protein-containing fractions were pooled, concentrated and injected on a 16/60 HiLoad Superdex 200 size exclusion column (GE Healthcare) equilibrated with a buffer containing 20 mM TRIS (pH 8.0), 100 mM NaCl and 5 mM DTT. Protein-containing fractions were pooled, concentrated to ∼20 mg ml^−1^ and flash frozen in liquid nitrogen for further use.

#### Purification of GST-tagged hsKap104, Kap104 and Acl4

Cleared cell lysate of proteins with N-terminal Glutathione-S-transferase (GST) tag was applied to a glutathione sepharose column equilibrated with a buffer containing 20 mM TRIS (pH 8.0), 100 mM NaCl and 5 mM DTT and eluted with a linear gradient of reduced glutathione. Pooled fractions were cleaved with PreScission protease (GE Healthcare) for at least 10 h and dialysed against a buffer containing 20 mM TRIS (pH 8.0), 100 mM NaCl and 5 mM DTT (GST-Kap104 and GST-Acl4 for subsequent GST pull-downs were dialysed but not treated with PreScission protease). Dialysed proteins were bound to a HiTrapQ HP (GE Healthcare) ion exchange column equilibrated in a buffer containing 20 mM TRIS (pH 8.0), 100 mM NaCl and 5 mM DTT and eluted with a linear salt gradient. Protein-containing fractions were pooled, concentrated and injected on a 16/60 HiLoad Superdex 200 size exclusion column (GE Healthcare) equilibrated with a buffer containing 20 mM TRIS (pH 8.0), 100 mM NaCl and 5 mM DTT. Protein-containing fractions were pooled, concentrated to ∼20 mg ml^−1^ and flash frozen in liquid nitrogen for further use.

#### *Purification of Ran* ^*Q69L*^

Cleared lysate of His_6_-Ran^Q69L^ was applied to a Ni-NTA column (Qiagen) equilibrated with a buffer containing 20 mM TRIS (pH 8.0), 500 mM NaCl and 5 mM β-ME and eluted with a linear imidazole gradient. Pooled fractions were cleaved with PreScission protease (GE Healthcare) for at least 10 h and dialysed against a buffer containing 20 mM TRIS (pH 8.0), 100 mM NaCl and 5 mM DTT. Cleaved protein was bound to a HiTrapQ HP (GE Healthcare) ion exchange column equilibrated in a buffer containing 20 mM TRIS (pH 8.0), 100 mM NaCl and 5 mM DTT and eluted with a linear salt gradient. Protein-containing fractions were pooled, concentrated and injected on a 16/60 HiLoad Superdex 75 size exclusion column (GE Healthcare) equilibrated with a buffer containing 20 mM TRIS (pH 8.0), 100 mM NaCl and 5 mM DTT. Purified Ran^Q69L^ was charged with GTP by incubation with 10 mM EDTA and 2 mM GTP for 30 min at 4 °C. Nucleotide exchange was stopped by the addition of 25 mM MgCl_2_ (ref. 23). Ran^Q69L^GTP was injected on a Superdex 200 10/300 GL size exclusion column (GE Healthcare) pre-equilibrated with a buffer containing 20 mM TRIS (pH 8.0), 100 mM NaCl, 5 mM DTT and 5 μM GTP. Protein-containing fractions were pooled, concentrated to ∼20 mg ml^−1^ and flash frozen in liquid nitrogen for further use.

#### *Purification of Acl4*·*RpL4*

His_6_-SUMO-tagged Acl4 and His_6_-SUMO-tagged RpL4, encompassing residues 28–361 and 1–277, respectively, were coexpressed, as previously described^10^. Filtered lysate was applied to a Ni-NTA column (Qiagen) equilibrated with a buffer containing 20 mM TRIS (pH 8.0), 500 mM NaCl and 5 mM β-ME and eluted with a linear imidazole gradient. Protein-containing fractions were pooled and cleaved with ULP1 and dialysed against a buffer containing 20 mM TRIS (pH 8.0), 100 mM NaCl and 5 mM β-ME. Dialysed proteins were applied to a Ni-NTA column equilibrated with a buffer containing 20 mM TRIS (pH 8.0), 100 mM NaCl and 5 mM β-ME and the unbound fraction was loaded onto a HiTrapQ HP (GE Healthcare) ion exchange column equilibrated in a buffer containing 20 mM TRIS (pH 8.0), 100 mM NaCl and 5 mM DTT and eluted with a linear salt gradient. Protein-containing fractions were pooled, concentrated and injected on a 16/60 HiLoad Superdex 200 size exclusion column (GE Healthcare) equilibrated with a buffer containing 20 mM TRIS (pH 8.0), 100 mM NaCl and 5 mM DTT. Protein-containing fractions were pooled, concentrated to ∼20 mg ml^−1^ and used for crystallization.

#### *Purification of hsKap104*·*RpL4* ^*EXT*^

*hs*Kap104 and RpL4^EXT^, encompassing residues 308–332, were purified individually, as described above. The *hs*Kap104·RpL4^EXT^ complex was assembled on a 16/60 HiLoad Superdex 200 size exclusion column (GE Healthcare) equilibrated with a buffer containing 20 mM TRIS (pH 8.0), 100 mM NaCl and 5 mM DTT. Complex assembly was carried out in the presence of a 5-fold molar excess of RpL4^EXT^ over *hs*Kap104 to yield a stoichiometric *hs*Kap104·RpL4^EXT^ complex. Protein-containing fractions were pooled, concentrated to ∼20 mg ml^−1^ and used for crystallization.

### Structure determination and refinement of Acl4·RpL4

Crystals of the *C. thermophilum* Acl4·RpL4 complex, encompassing residues 28–361 and 1–277, respectively, were obtained by hanging drop vapour diffusion at 21 °C using 1 μl protein solution and 1 μl reservoir solution containing 0.1 M BIS–TRIS (pH 5.5), 2% (v/v) Tacsimate (pH 5.5), and 13% (w/v) PEG 3350. Acl4·RpL4 crystals grew in the orthorhombic space group P2_1_2_1_2 at a protein concentration of 17.5 mg ml^−1^ and reached their maximum size of ∼100 × 50 × 50 μm^3^ within one week. Cryo protection of the crystals was achieved with 0.1 M BIS-TRIS (pH 5.5), 2% (v/v) Tacsimate (pH 5.5), 15% (w/v) PEG 3350 and 20% (v/v) ethylene glycol added in 5% increments. Collection of X-ray diffraction data was performed at 100 K at beamline BL12-2 at the Stanford Synchrotron Radiation Lightsource (SSRL) and crystals diffracted to a resolution of 2.4 Å. X-ray data were processed using XDS (ref. 24). The structure of the Acl4·RpL4 complex was solved by single-wavelength anomalous dispersion (SAD) using anomalous scattering data collected at the selenium edge of SeMet-labelled crystals. Eight selenium sites were identified with SHELXD and initial phases were calculated with SHARP^25^,^26^. Density modification with solvent flattening and histogram matching was performed using DM (ref. 27). The initial electron density map was of high-quality and allowed for building of a complete model of the Acl4·RpL4 complex. A final model of the complex was generated by iterative rounds of model building and refinement in Coot and PHENIX, consisting of Acl4 residues 28–361 and RpL4 residues 4–272 (refs 28, 29). No electron density was observed for RpL4 residues 1–3, 78–88, 189–202 and 273–277, which are presumed to be disordered. The structure was refined to *R*_work_ and *R*_free_ values of 19.1% and 22.7%, respectively. The Acl4·RpL4 model possesses excellent stereochemical parameters with no residues in disallowed regions of the Ramachandran plot as determined by MolProbity^30^. Further details of the data collection and refinement statistics are summarized in Table 1.

### Structure determination and refinement of *hs*Kap104·RpL4^EXT^

Crystals of the *hs*Kap104·RpL4^EXT^ complex were obtained by hanging drop vapour diffusion at 21 °C using 1 μl protein solution and 1 μl reservoir solution containing 0.1 M BIS-TRIS-propane (pH 7.0) and 0.5 M sodium citrate tribasic. *hs*Kap104·RpL4^EXT^ crystals grew in the orthorhombic space group P2_1_2_1_2 at a protein concentration of 5 mg ml^−1^ and reached their maximum size of ∼100 × 50 × 50 μm^3^ within one week. Cryo protection of the crystals was achieved with 0.1 M BIS-TRIS-propane (pH 7.0) and 0.5 M sodium citrate tribasic and 24% (v/v) glycerol added in 8% increments. Collection of X-ray diffraction data was performed at 100 K at beamline BL12-2 at the Stanford Synchrotron Radiation Lightsource (SSRL) and crystals diffracted to a resolution of 3.0 Å. X-ray data were processed using XDS (ref. 24). The structure of the *hs*Kap104·RpL4^EXT^ complex was solved by molecular replacement using the coordinates of *hs*Kap-β2 (PDB ID 4JLQ) as a search model in phaser^28^,^31^. The initial electron density map was of high quality and allowed for building of a complete model of the Kap104·RpL4^EXT^ complex. A final model of the complex was generated by iterative rounds of model building and refinement in Coot and PHENIX, consisting of *hs*Kap104 residues 1–890 and RpL4^EXT^ residues 326–332 (refs 28, 29). No electron density was observed for *hs*Kap104 residues 1–4 and 321–367 and for RpL4^EXT^ residues 308–325, which are presumed to be disordered. The structure was refined to *R*_work_ and *R*_free_ values of 20.8% and 23.8%, respectively. The *hs*Kap104·RpL4^EXT^ model possesses excellent stereochemical parameters with no residues in the disallowed regions of the Ramachandran plot as determined by MolProbity^30^. Further details of the data collection and refinement statistics are summarized in Table 1.

### GST pull-down interaction analysis

Interaction studies with GST-Acl4 coexpressed with SUMO-RpL4^ΔEXT^ were performed using GST pull-down experiments. Approximately 100 μl glutathione-coupled sepharose beads (GE Healthcare) were equilibrated with a buffer containing 20 mM TRIS (pH 8.0), 100 mM NaCl and 5 mM DTT and were incubated with cleared and filtered lysate from 1 l bacterial expression culture for 1 h at 4 °C. GST-beads were washed three times with 15 ml buffer containing 20 mM TRIS (pH 8.0), 100 mM NaCl and 5 mM DTT and centrifuged with 500*g* at 4 °C. Bound protein was eluted from the beads with 250 μl buffer containing 20 mM TRIS (pH 8.0), 100 mM NaCl, 5 mM DTT and 25 mM reduced glutathione. Eluted protein was resolved on a 12.5% SDS–polyacrylamide gel electrophoresis (SDS–PAGE) gel followed by visualization with Coomassie Brilliant Blue staining.

For GST pull-down experiments with pre-purified proteins, 20 μl of glutathione-coupled sepharose beads were equilibrated with a buffer containing 20 mM TRIS (pH 8.0), 100 mM NaCl and 5 mM DTT and incubated for 1 h with 35 nmol GST-tagged bait proteins and untagged prey proteins. After incubation, the beads were washed four times with 200 μl buffer containing 20 mM TRIS (pH 8.0), 100 mM NaCl and 5 mM DTT and centrifuged with 500*g* at 4 °C. SDS-sample buffer was added to the beads, followed by boiling at 95 °C for 5 min and centrifugation at 30,000*g* for 5 min. Samples were resolved on a 12.5% SDS–PAGE gel and visualized with Coomassie Brilliant Blue staining.

### Size exclusion chromatography interaction analysis

Purified Acl4, RpL4, Kap104 and Kap-α variants were analysed by size exclusion chromatography (SEC). Samples were injected on a Superdex 200 10/300 GL size exclusion column (GE Healthcare) pre-equilibrated with a buffer containing 20 mM TRIS (pH 8.0), 100 mM NaCl and 5 mM DTT. Pre-incubation was performed for 1 h at 4 °C before injection on a size exclusion column. Protein containing fractions were resolved on a 12.5% SDS–PAGE gel and visualized with Coomassie Brilliant Blue staining.

### Yeast analysis

All yeast media was prepared and Lithium-acetate driven *S. cerevisiae* transformations were performed according to standard protocols. The *S. cerevisiae acl4Δ* strain was generated by replacing the Acl4 gene with a *kanMX4* cassette by homologous recombination, as previously described^32^. Details of yeast expression vectors are summarized in Supplementary Table 2.

### Growth analysis and fluorescence microscopy

The growth analysis was performed in *S. cerevisiae acl4Δ* strains that were transformed with pRS415 constructs carrying various mCherry-tagged Acl4 variants. Transformed cells were selected twice on SDC-LEU plates, before analysis. Ten-fold dilution series were prepared and 17.5 μl were spotted on SDC-LEU plates and grown at 23, 30 and 37 °C for 2–3 days. Localization assays were performed using pRS415 vectors carrying mCherry-tagged Acl4 variants and a pRS413 vector harbouring eGFP-tagged RpL4. Transformed cells were selected twice on SDC-LEU-HIS plates before analysis. The variants were grown in SDC-LEU-HIS medium at 30 °C to mid-log phase. For heat-shock analysis, cells were grown at 30 °C to mid-log phase before shifting cells to 37 °C for 6 h. For fluorescence microscopy 1 ml of cells was centrifuged at 500*g* and washed once with 1 ml of water. The cell pellet was resuspended in 100 μl water and 10 μl were analysed using a Carl Zeiss Observer Z.1 equipped with a Hamamatsu C10600 Orca-R2 camera.

### Western blot analysis

*In vivo* Acl4 expression levels were tested by transformation of *S. cerevisiae acl4Δ* strains with pRS415 constructs carrying various HA-mCherry-tagged Acl4 variants. Transformed cells were selected twice on SDC-LEU plates, before analysis. Protein extraction from cells was performed via NaOH and TCA treatment^33^. Specifically, cells were grown at 30 °C to an OD of ∼1.0 before harvesting of 1 ml of culture. Cell pellets were resuspended and vortexed in 1 ml of a solution containing 1.85 M NaOH and 7.4% (v/v) β-ME before incubation for 10 min on ice. Proteins were precipitated by addition of 150 μl of 50% (w/v) TCA and incubation for 10 min on ice, followed by centrifugation at 30,000*g* for 2 min. The pellet was washed twice with 1 ml of ice-cold acetone and air-dried at room temperature before resuspension in SDS loading buffer. Western blot analysis was performed with a rabbit anti-hexokinase antibody (US Biological; H2035-02; 1:10,000 dilution), an anti-rabbit antibody fused to an IR800 fluorescent probe (Licor, 926-32211; 1:5,000 dilution), a mouse anti-HA antibody (Covance; MMS-101P; 1:5,000 dilution), and an anti-mouse antibody coupled to alkaline phosphatase (Promega; S3721; 1:5,000 dilution). Antibodies were diluted in TBS-T supplemented with 5% (w/v) milk powder and washes were carried out in TBS-T.

### Animation of Acl4·RpL4 disassembly

Acl4 *apo* (PDB ID 4YNV) and ribosome-bound RpL4 (PDB ID 4V88) were superposed with Acl4·RpL4 structure as reference^10^,^17^. Morphing of the Acl4·RpL4 complex into the open Acl4 *apo* state and ribosome-bound RpL4 was animated using PyMOL (www.pymol.org).

### Illustrations and figures

Size exclusion chromatography profiles were generated with IGOR (WaveMetrics) and assembled with Adobe Illustrator. All structure figures were generated with PyMOL (www.pymol.org). Sequence alignments were generated using ClustalX and coloured with ALSCRIPT^34^,^35^. Electrostatic potentials were calculated with APBS (Adaptive Poisson-Boltzmann Solver) software^36^.

### Data availability

The coordinates and structure factors have been deposited with the Protein Data Bank with accession codes 5TQB (Acl4·RpL4) and 5TQC (Kap104·RpL4^EXT^). The data that support the findings of this study are available from the corresponding author on request.

## Additional information

**How to cite this article:** Huber, F. M. & Hoelz, A. Molecular basis for protection of ribosomal protein L4 from cellular degradation. *Nat. Commun.* **8,** 14354 doi: 10.1038/ncomms14354 (2017).

**Publisher's note:** Springer Nature remains neutral with regard to jurisdictional claims in published maps and institutional affiliations.

## Supplementary Material

Supplementary Information Supplementary Figures, Supplementary Tables and Supplementary References

Supplementary Movie 1 Rotating Acl4•RpL4 in cartoon representation. Acl4•RpL4 is colored according to Fig. 1.

Supplementary Movie 2 Morphing of the Acl4•RpL4 complex to the conformations of Acl4 apo and RpL4 as found in the 60S large ribosomal subunit. Acl4 and RpL4 are shown in cartoon representation and colored according to Fig. 1.

## Figures and Tables

**Table 1:** Data collection and refinement statistics.

*Data Collection*		
Protein	Acl4^28–361^·RpL4^1-277^	*hs*Kap104·RpL4^308–332^
PDB ID	5TQB	5TQC
Synchrotron	SSRL*	SSRL*
Beamline	12–2	12–2
Space group	P2_1_2_1_2	P2_1_2_1_2
Cell dimensions		
*a*, *b*, *c* (Å)	121.0, 127.9, 42.7	68.6, 130.7, 174.2
α, β, γ (°)	90.0, 90.0, 90.0	90.0, 90.0, 90.0
	*Se Peak*	*Native*
Wavelength (Å)	0.9792	1.0000
Resolution (Å)	50.0–2.4	50.0–3.0
*R*_merge_ (%)†	8.9 (99.0)	9.3 (192.7)
*R*_pim_ (%)†	2.6 (28.5)	2.7 (53.5)
*<I*>/<σ*I>*†	13.6 (1.9)	20.7 (1.6)
CC_1/2_ †	99.9 (89.8)	99.9 (75.7)
Completeness (%)†	99.1 (99.1)	99.8 (99.9)
No. of observations	338,722	425,167
No. of unique reflections†	49,797 (8,039)	32,119 (5,078)
Redundancy†	6.8 (6.6)	13.2 (13.7)

*Refinement*		
Resolution (Å)	50.0–2.4	50.0–3.0
No. of reflections	49,767	32,065
No. of reflections test set	2,505 (5.0%)	1,606 (5.0%)
*R*_work_/*R*_free_ (%)	19.1/22.7	20.8/23.8
No. atoms	4,605	6,750
Protein	4,510	6,750
Ligands	39	0
Water	56	0
*B*-factors		
Protein	73	103
Ligands	87	N/A
Water	60	N/A
r.m.s.d.		
Bond lengths (Å)	0.004	0.002
Bond angles (°)	0.7	0.7

*Ramachandran plot*‡		
Favored (%)	96.7	97.4
Outliers (%)	0.0	0.0

*MolProbity*		
Clash score‡	1.98	1.77
MolProbity score‡	1.17	1.05

Crystallographic analysis.

^*^SSRL, Stanford Synchrotron Radiation Lightsource.

^†^Highest-resolution shell is shown in parentheses.

^‡^As determined by MolProbity^30^.
