# Supplementary material for: Molecular basis for protection of ribosomal protein L4 from cellular degradation
Source: Nat Commun. 2017 Feb 2;8:14354. doi: 10.1038/ncomms14354 (PMC5296656; doi:10.1038/ncomms14354)
Supplement: Supplementary Information — Supplementary Figures, Supplementary Tables and Supplementary References [file ncomms14354-s1.pdf]

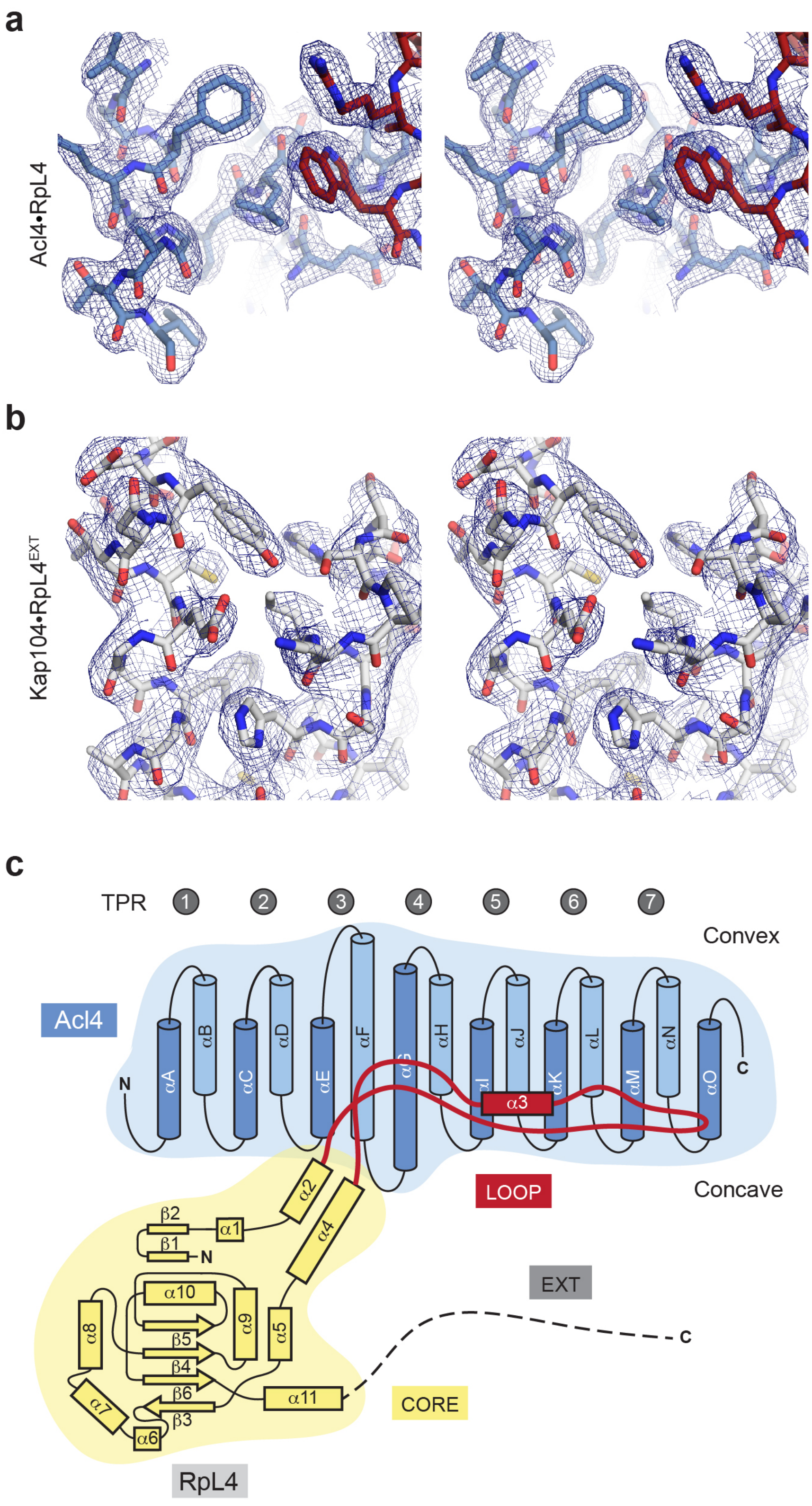

Supplementary Figure 1

Supplementary Figure 1

Representations of the Acl4•RpL4 and Kap104•RpL4^{EXT} crystal structures.

(**a**) Stereo view of a stick representation of Acl4•RpL4 with a section of the final $2|F_o|-|F_c|$ electron density map contoured at 1.0 σ.

(**b**) Stereo view of a stick representation of Kap104•RpL4^{EXT} with a section of the final $2|F_o|-|F_c|$ electron density map contoured at 1.0 σ.

(**c**) The architecture of the Acl4•RpL4 complex is represented by the schematic arrangement of its secondary structure elements. The coloring is according to the color scheme in Fig. 1a.

Acl4

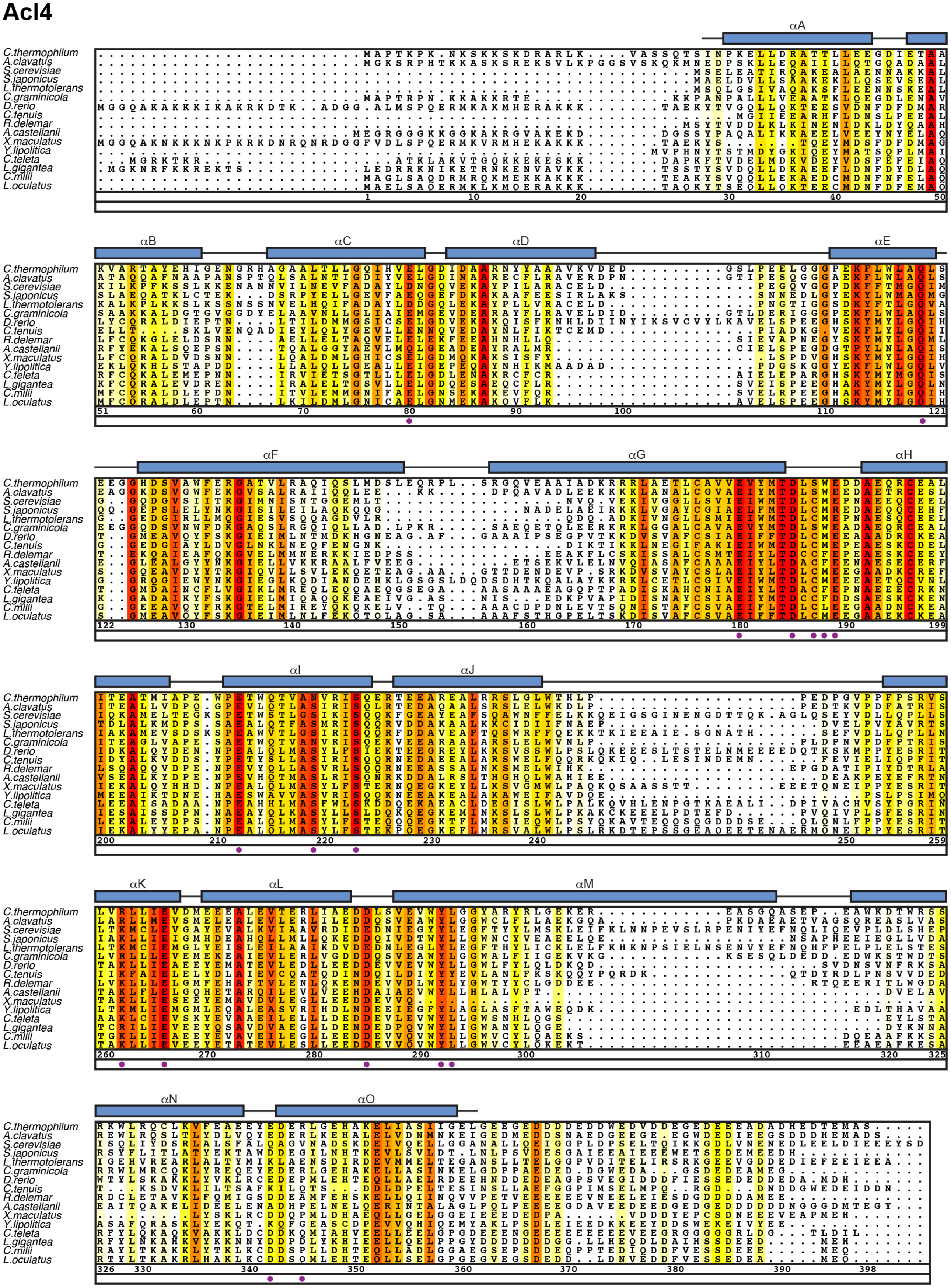

Supplementary Figure 2

Supplementary Figure 2

Multispecies sequence alignment of Acl4.

Protein sequences of Acl4 from 16 species were aligned and colored according to a BLOSUM62 matrix. The sequence conservation is represented in shading from white (< 40 % similarity), yellow (40 % similarity) to dark red (100 % identity). Numbering of the alignment below is relative to the *C. thermophilum* Acl4 protein sequence. The secondary structure of Acl4 as observed in the Acl4•RpL4 structure is shown in blue rectangles, representing α-helices (αA to αO) and gray lines indicate coil regions. Purple dots indicate Acl4 surface residues that were mutated in this study.

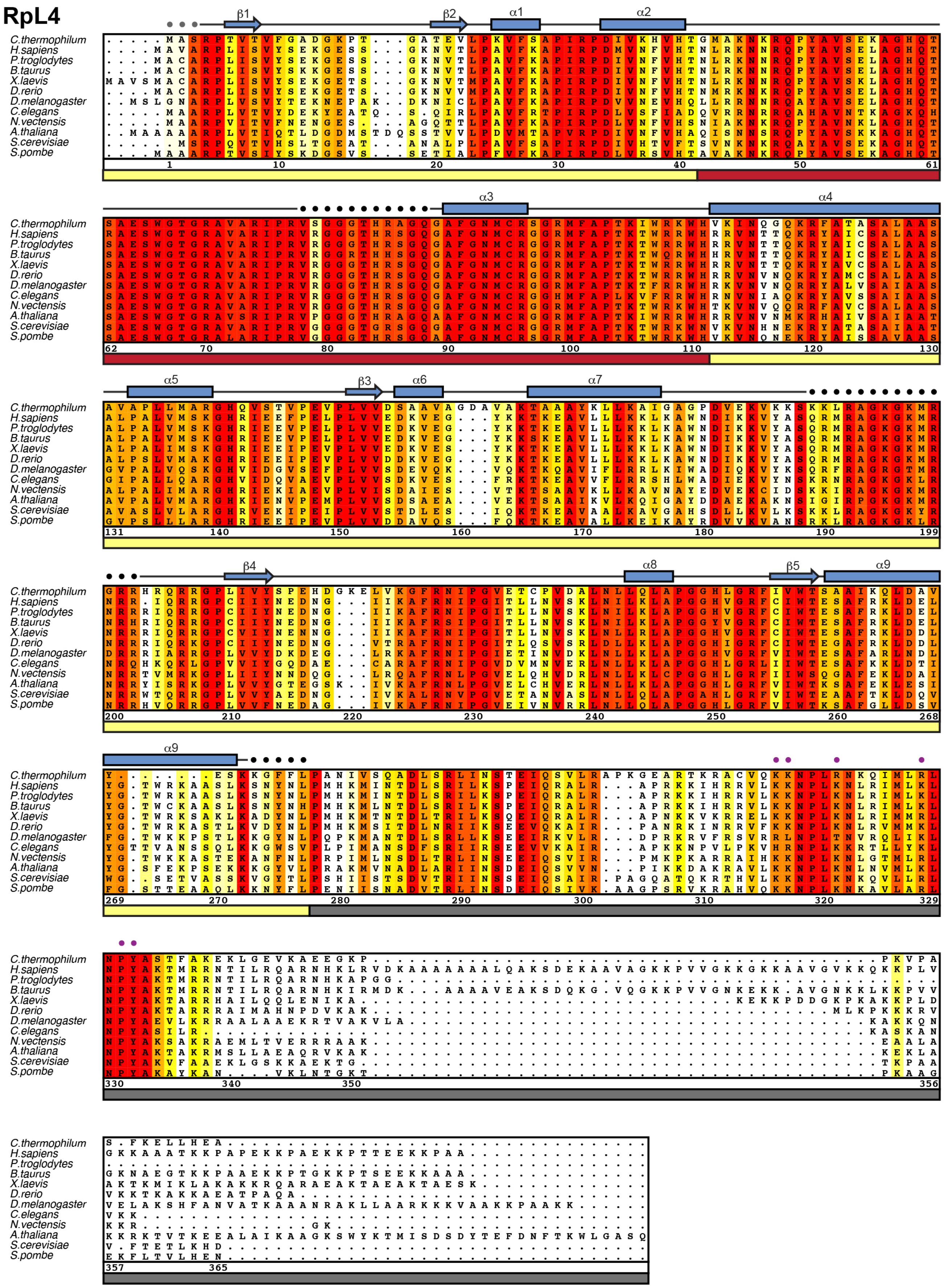

Supplementary Figure 3

Supplementary Figure 3

Multispecies sequence alignment of RpL4.

Protein sequences of RpL4 from 12 species were aligned and colored according to a BLOSUM62 matrix. The sequence conservation is represented in shading from white (< 50 % similarity), yellow (50 % similarity) to dark red (100 % identity). Numbering of the alignment is relative to *C. thermophilum* RpL4. The secondary structure of RpL4 as observed in the Acl4•RpL4 crystal structure is illustrated with arrows and rectangles, representing beta-strands (β1 to β5) and α-helices (α1 to α9), respectively, and colored according to Fig. 1a. Gray dots indicate residues that were part of the crystallization construct but were not observed in the final electron density map and thus are presumed to be disordered. Purple dots indicate $RpL4^{EXT}$ residues that were mutated in this study. Residues corresponding to $RpL4^{CORE}$, $RpL4^{LOOP}$, and $RpL4^{EXT}$ are highlighted by a yellow, red and gray bar, respectively.

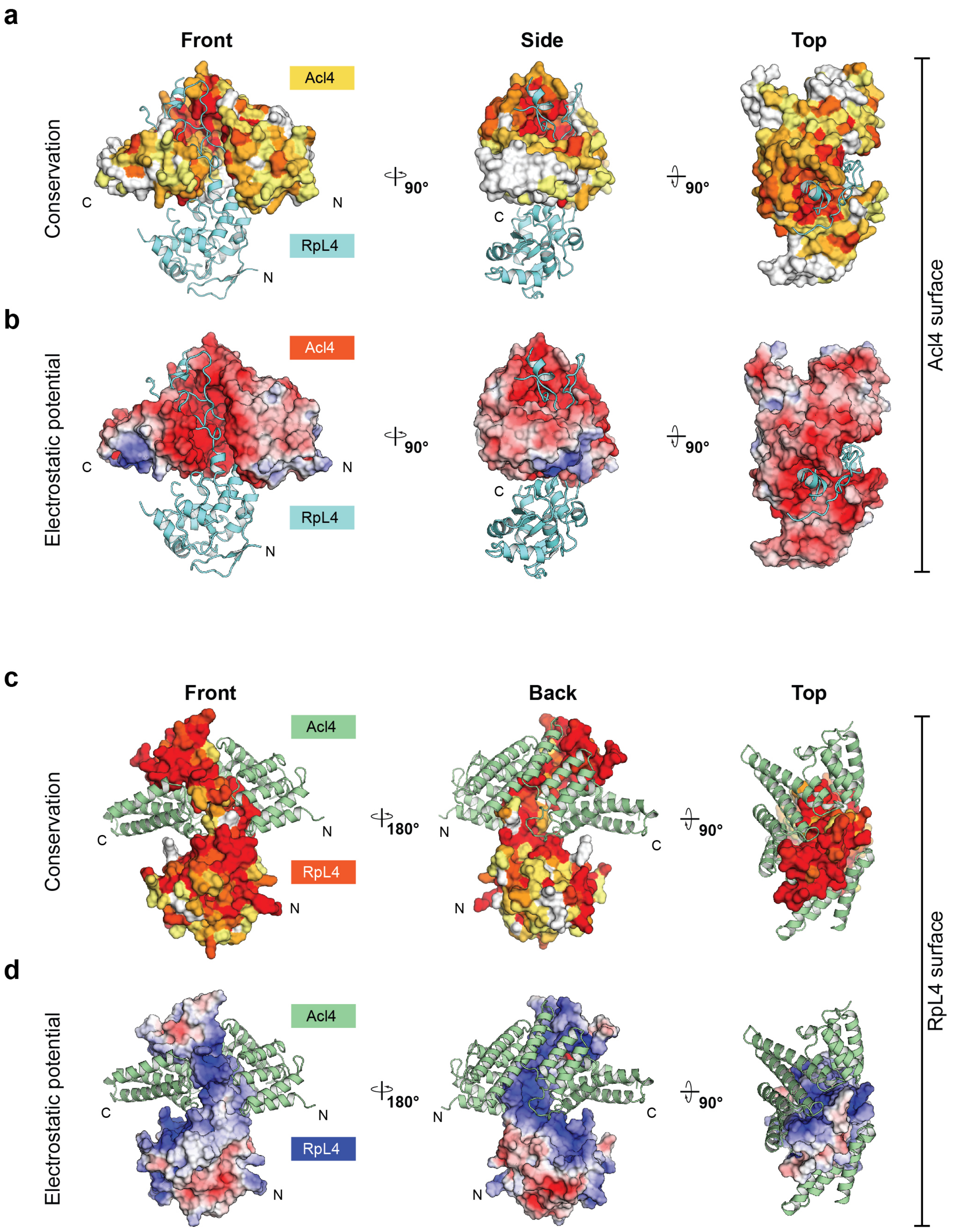

Supplementary Figure 4

Supplementary Figure 4

Conservation and electrostatic surface analysis of the Acl4•RpL4 crystal structure.

(**a**) Surface representations of Acl4 colored according to the multi-species sequence alignment shown in Supplementary Fig. 2. RpL4 is shown in cartoon representation and colored in teal. The Acl4•RpL4 complex is shown from the front, side and top. (**b**) Surface representations of Acl4 colored according to its electrostatic surface potential from -5 $k_B T/e$ (red) to +5 $k_B T/e$ (blue). RpL4 is shown in cartoon representation and colored in teal. (**c**) Surface representation of RpL4 colored according to the multi-species sequence alignment shown in Supplementary Fig. 3. Acl4 is shown in cartoon representation and colored in green. (**d**) Surface representations of RpL4 colored according to its electrostatic surface potential from -5 $k_B T/e$ (red) to +5 $k_B T/e$ (blue).

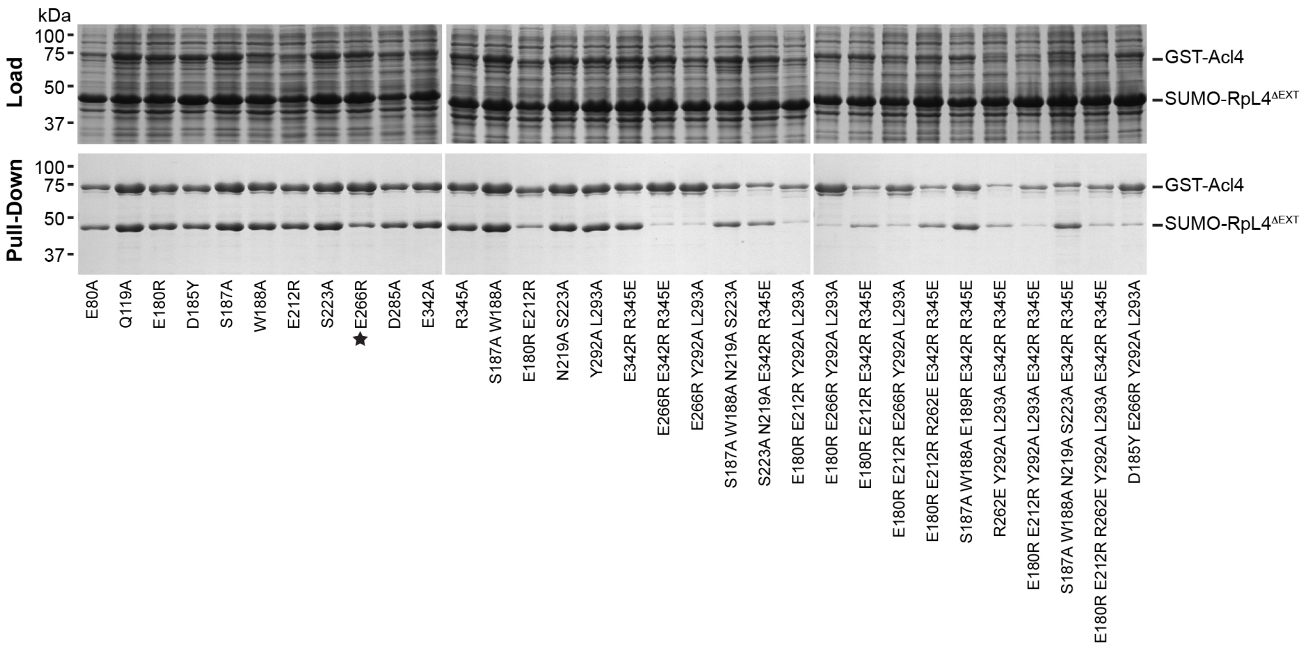

Supplementary Figure 5

Supplementary Figure 5

Interaction analysis of *C. thermophilum* Acl4 surface mutations.

GST pull-down interaction analysis of His_6-SUMO-RpL4$^{\Delta EXT}$ and GST-Acl4 variants. Samples were resolved on SDS-PAGE gels and visualized by Coomassie Brilliant Blue staining. SDS-PAGE gels on top and bottom show the loaded soluble fraction that was incubated with glutathione beads and eluted pulled-down samples, respectively.

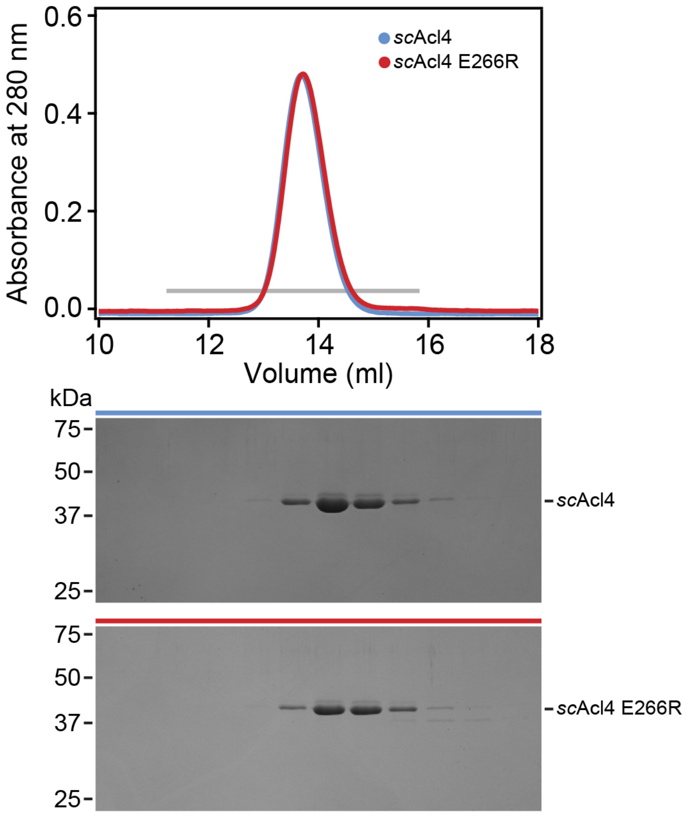

Supplementary Figure 6

Supplementary Figure 6

Biochemical analysis of the *sc*Acl4 E266R mutant

Size exclusion chromatography (SEC) analysis of the *sc*Acl4 E266R mutant. The absorbance at 280 nm is plotted against the elution volume of a Superdex 200 10/300 GL size exclusion column. Fractions indicated by a gray bar were resolved by SDS-PAGE gel and visualized by Coomassie Brilliant Blue staining.

a

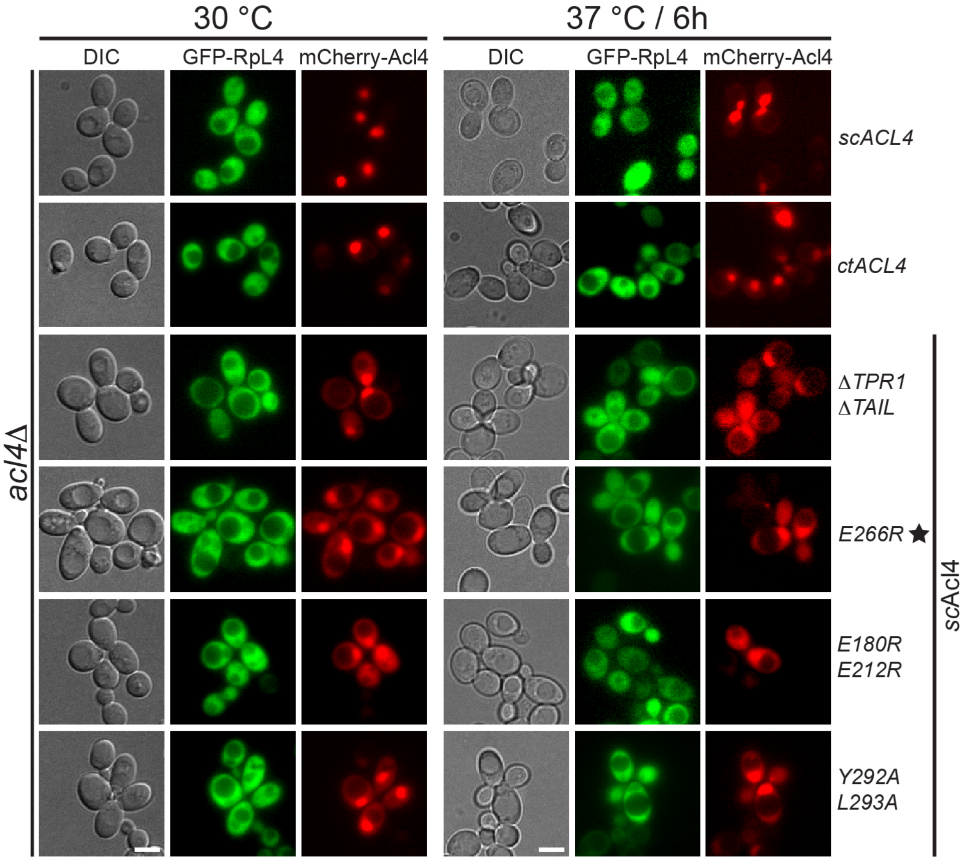

b

Fig. 2g, original Western blot

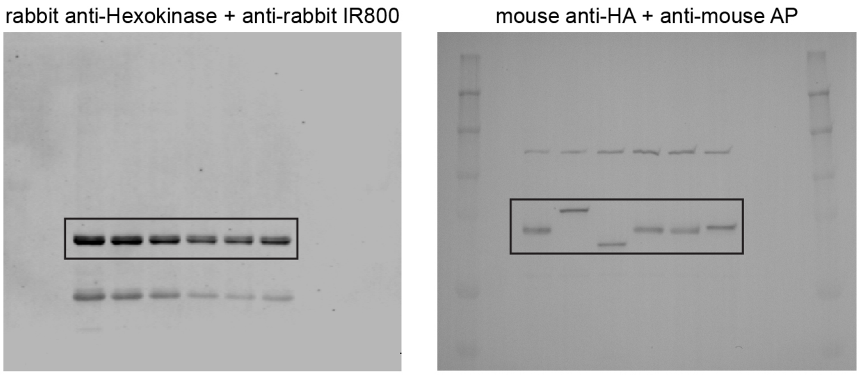

Supplementary Figure 7

Supplementary Figure 7

In vivo localization analysis of Acl4 and RpL4.

(**a**) Subcellular localization analysis of mCherry-tagged Acl4 variants (red) and eGFP-tagged RpL4 (green) in a *S. cerevisiae acl4Δ* strain. Differential interference contrast (DIC) images are shown in gray scale. The left panel represents yeast grown to mid-log phase at 30 °C, while the right panel shows cells that were grown to mid-log phase at 30 °C and then shifted to 37 °C for 6 hours. Scale bars are 5 μm. (**b**) Original uncropped Western blots of Fig. 2g. Black boxes indicate the cropped sections of the same membrane probed and visualized with different antibodies.

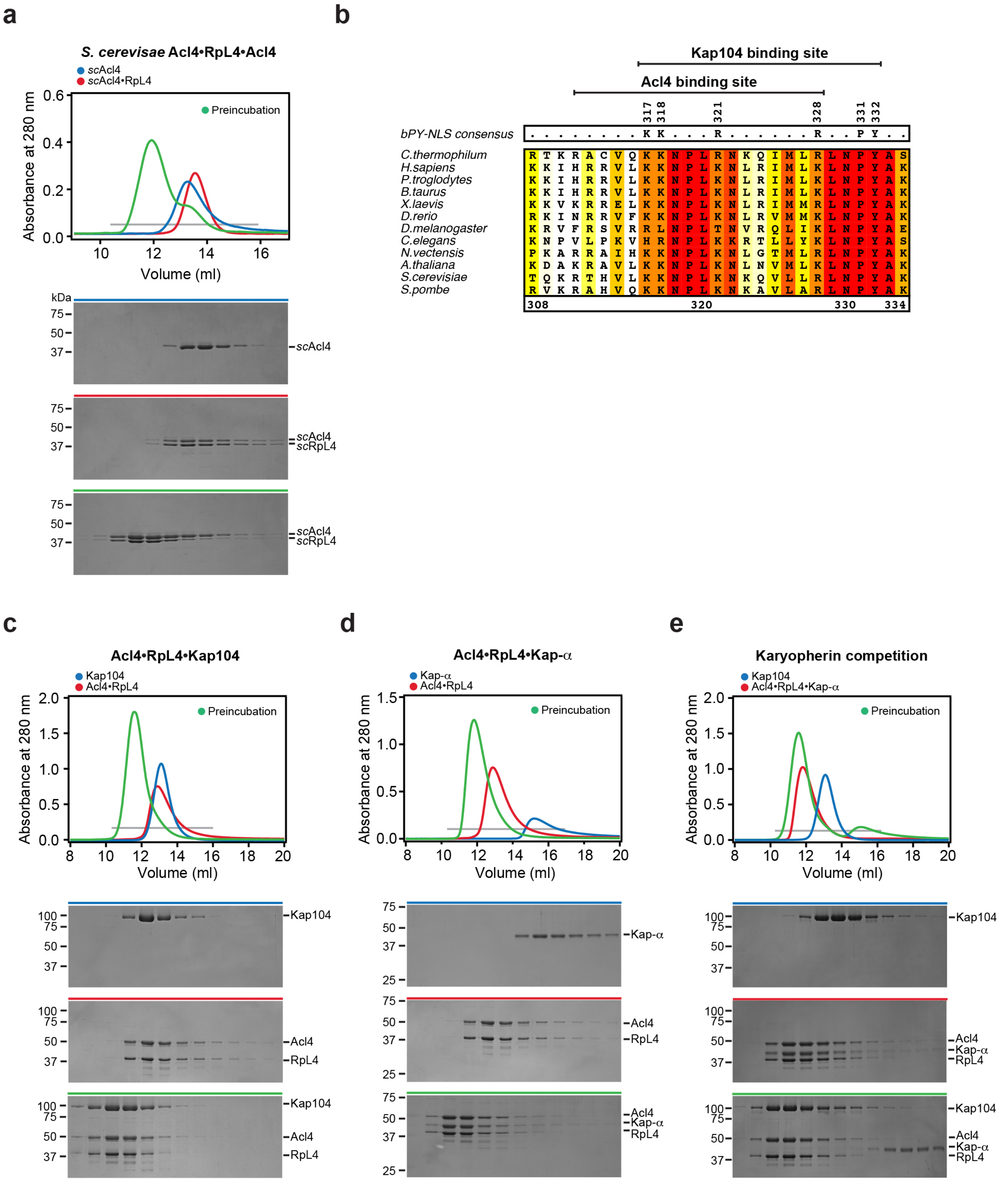

Supplementary Figure 8

Supplementary Figure 8

Biochemical analysis of $RpL4^{EXT}$ interaction partners.

(**a**) Size exclusion chromatography (SEC) analysis of the *S. cerevisiae* Acl4•RpL4•Acl4 complex. The absorbance at 280 nm is plotted against the elution volume of a Superdex 200 10/300 GL size exclusion column. (**b**) Protein sequences of RpL4 from 12 species were aligned and colored as in Supplementary Fig. 3. The consensus sequence of the basic PY-NLS is shown above the alignment. Numbering below the alignment is relative to *C. thermophilum* RpL4. Kap104 and Acl4 binding sites are indicated with black bars. (**c**) SEC analysis of the Acl4•RpL4•Kap104 complex. (**d**) SEC analysis of the Acl4•RpL4•Kap-α complex. (**e**) SEC analysis of a preformed Acl4•RpL4•Kap-α complex incubated with additional Kap104. Fractions indicated by a gray bar were resolved by SDS-PAGE gel and visualized by Coomassie Brilliant Blue staining.

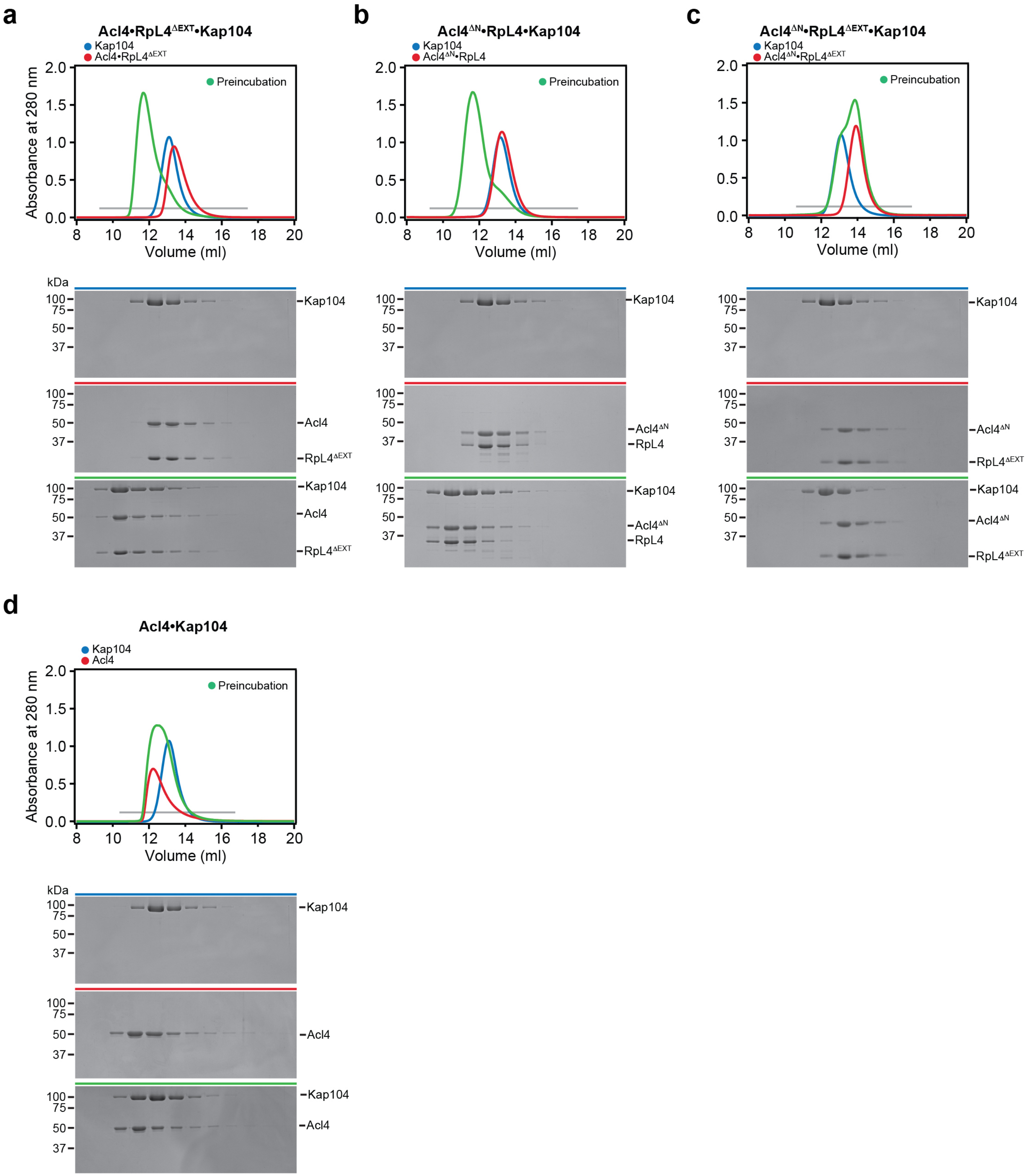

Supplementary Figure 9

Supplementary Figure 9

Kap104 interaction with Acl4•RpL4.

(**a**) Size exclusion chromatography (SEC) analysis of the Acl4•RpL4$^{\Delta EXT}$•Kap104 complex. The absorbance at 280 nm is plotted against the elution volume of a Superdex 200 10/300 GL size exclusion column. (**b**) SEC analysis of the Acl4$^{\Delta N}$•RpL4•Kap104 complex. (**c**) SEC analysis of the Acl4$^{\Delta N}$•RpL4$^{\Delta EXT}$•Kap104 complex. (**d**) SEC analysis of Acl4•Kap104. The elution profile and SDS-PAGE gel of Kap104 is included in panels **a**-**d** as reference point for Kap104 elution. Fractions indicated by a gray bar were resolved by SDS-PAGE gel and visualized by Coomassie Brilliant Blue staining.

Supplementary Table 1: Yeast expression constructs

Plasmid	Protein	Residues (Mutations)	Vector	Restriction sites 5', 3'	Selection marker
pRS413-P_{Nop1}-eGFP-*sc*RPL4	*sc*RpL4	1-362	pRS413	BamHI, NotI	*HIS3*
pRS415-P_{Nop1}-mCherry-*scACL4*	*sc*Acl4	1-387	pRS415	BamHI, NotI	*LEU2*
pRS415-P_{Nop1}-mCherry-*scacl4 ΔTPR1ΔTAIL*	*sc*Acl4	40-372	pRS415	BamHI, NotI	*LEU2*
pRS415-P_{Nop1}-mCherry-*scacl4 E266R*[a]	*sc*Acl4	1-387 (E266R; E236R in *S. cerevisiae*)	pRS415	BamHI, NotI	*LEU2*
pRS415-P_{Nop1}-mCherry-*scacl4 E180R E212R*[a]	*sc*Acl4	1-387 (E180R E212R; E131R E164R in *S. cerevisiae*)	pRS415	BamHI, NotI	*LEU2*
pRS415-P_{Nop1}-mCherry-*scacl4 Y292A L293A*[a]	*sc*Acl4	1-387 (Y292A L293A; Y262A L263A in *S. cerevisiae*)	pRS415	BamHI, NotI	*LEU2*
pRS415-P_{Nop1}-mCherry-*ctACL4*	*sc*Acl4	1-398	pRS415	BamHI, NotI	*LEU2*
pRS415-P_{Nop1}-HA-mCherry-*scACL4*	*sc*Acl4	1-387	pRS415	BamHI, NotI	*LEU2*
pRS415-P_{Nop1}-HA-mCherry-*scacl4 ΔTPR1ΔTAIL*	*sc*Acl4	40-372	pRS415	BamHI, NotI	*LEU2*
pRS415-P_{Nop1}-HA-mCherry-*scacl4 E266R*[a]	*sc*Acl4	1-387 (E266R; E236R in *S. cerevisiae*)	pRS415	BamHI, NotI	*LEU2*
pRS415-P_{Nop1}-HA-mCherry-*scacl4 E180R E212R*[a]	*sc*Acl4	1-387 (E180R E212R; E131R E164R in *S. cerevisiae*)	pRS415	BamHI, NotI	*LEU2*
pRS415-P_{Nop1}-HA-mCherry-*scacl4 Y292A L293A*[a]	*sc*Acl4	1-387 (Y292A L293A; Y262A L263A in *S. cerevisiae*)	pRS415	BamHI, NotI	*LEU2*
pRS415-P_{Nop1}-HA-mCherry-*ctACL4*	*sc*Acl4	1-398	pRS415	BamHI, NotI	*LEU2*
pRS415-P_{Nop1}-mCherry[b]	N/A	N/A	pRS415	N/A	*LEU2*

[a]Mutants are listed with both *C. thermophilum* and the corresponding *S. cerevisiae* numbering

[b]Sikorski et al., 1989

Supplementary Table 2: Bacterial expression constructs

Protein	Residues (Mutations)	Expression Vector	Restriction Sites 5', 3'	N-terminal overhang
*ct*Acl4	1-398	pET28b-SUMO	BamHI, NotI	S
*ct*Acl4	1-398	pGEX-6P-1	BamHI, NotI	GMGS
*ct*Acl4	1-398 (E80A)	pGEX-6P-1	BamHI, NotI	GMGS
*ct*Acl4	1-398 (Q119A)	pGEX-6P-1	BamHI, NotI	GMGS
*ct*Acl4	1-398 (D185Y)	pGEX-6P-1	BamHI, NotI	GMGS
*ct*Acl4	1-398 (E180R)	pGEX-6P-1	BamHI, NotI	GMGS
*ct*Acl4	1-398 (S187A)	pGEX-6P-1	BamHI, NotI	GMGS
*ct*Acl4	1-398 (W188A)	pGEX-6P-1	BamHI, NotI	GMGS
*ct*Acl4	1-398 (E212R)	pGEX-6P-1	BamHI, NotI	GMGS
*ct*Acl4	1-398 (E266R)	pGEX-6P-1	BamHI, NotI	GMGS
*ct*Acl4	1-398 (D285A)	pGEX-6P-1	BamHI, NotI	GMGS
*ct*Acl4	1-398 (E342R)	pGEX-6P-1	BamHI, NotI	GMGS
*ct*Acl4	1-398 (R345E)	pGEX-6P-1	BamHI, NotI	GMGS
*ct*Acl4	1-398 (E180R E212R)	pGEX-6P-1	BamHI, NotI	GMGS
*ct*Acl4	1-398 (S187A W188A)	pGEX-6P-1	BamHI, NotI	GMGS
*ct*Acl4	1-398 (N219A S223A)	pGEX-6P-1	BamHI, NotI	GMGS
*ct*Acl4	1-398 (Y292A L292A)	pGEX-6P-1	BamHI, NotI	GMGS
*ct*Acl4	1-398 (E342R R345E)	pGEX-6P-1	BamHI, NotI	GMGS
*ct*Acl4	1-398 (E266R Y292A L292A)	pGEX-6P-1	BamHI, NotI	GMGS
*ct*Acl4	1-398 (E266R E342R R345E)	pGEX-6P-1	BamHI, NotI	GMGS
*ct*Acl4	1-398 (S187A W188A N219A S223A)	pGEX-6P-1	BamHI, NotI	GMGS
*ct*Acl4	1-398 (N219A S223A E342R R345E)	pGEX-6P-1	BamHI, NotI	GMGS
*ct*Acl4	1-398 (E180R E212R Y292A L292A)	pGEX-6P-1	BamHI, NotI	GMGS
*ct*Acl4	1-398 (E180R E212R E342R R345E)	pGEX-6P-1	BamHI, NotI	GMGS
*ct*Acl4	1-398 (E180R E266R Y292A L292A)	pGEX-6P-1	BamHI, NotI	GMGS
*ct*Acl4	1-398 (D185Y E266R Y292A L293A)	pGEX-6P-1	BamHI, NotI	GMGS
*ct*Acl4	1-398 (E180R E212R E266R Y292A L292A)	pGEX-6P-1	BamHI, NotI	GMGS
*ct*Acl4	1-398 (E180R E212R R262E E342R R345E)	pGEX-6P-1	BamHI, NotI	GMGS
*ct*Acl4	1-398 (S187A W188A E189R E342R R345E)	pGEX-6P-1	BamHI, NotI	GMGS
*ct*Acl4	1-398 (R262E Y292A L293A E342R R345E)	pGEX-6P-1	BamHI, NotI	GMGS
*ct*Acl4	1-398 (E180R E212R Y292A L292A E342R R345E)	pGEX-6P-1	BamHI, NotI	GMGS
*ct*Acl4	1-398 (S187A W188A N219A S223A E342R R345E)	pGEX-6P-1	BamHI, NotI	GMGS
*ct*Acl4	1-398 (E180R E212R R262E Y292A L292A E342R R345E)	pGEX-6P-1	BamHI, NotI	GMGS
*ct*Acl4	28-398	pET28a-SUMO	BamHI, NotI	S
*ct*Acl4[a]	28-361	pET28a-SUMO	BamHI, NotI	S
*ct*Acl4	28-338	pET28a-SUMO	BamHI, NotI	S
*sc*Acl4	1-387	pET28a-SUMO	BamHI, NotI	S
*sc*Acl4	1-387	pGEX-6P-1	BamHI, NotI	GMGS
*sc*Acl4[b]	1-387 (E266R; E236R in *S. cerevisiae*)	pET28a-SUMO	BamHI, NotI	S
*sc*Acl4[b]	1-387 (E180R E212R; E131R E164R in *S. cerevisiae*)	pET28a-SUMO	BamHI, NotI	S
*sc*Acl4[b]	1-387 (Y292A L293A; Y262A L263A in *S. cerevisiae*)	pET28a-SUMO	BamHI, NotI	S
*sc*Acl4	40-372	pGEX-6P-1	BamHI, NotI	GMGS
*ct*RpL4	1-365	pETDuet1-SUMO	BamHI, NotI	S
*ct*RpL4	1-365 (K316A, K317A, R321A)	pETDuet1-SUMO	BamHI, NotI	S
*ct*RpL4	1-365 (R328A)	pETDuet1-SUMO	BamHI, NotI	S
*ct*RpL4	1-365 (P331A, Y332A)	pETDuet1-SUMO	BamHI, NotI	S
*ct*RpL4	1-353	pETDuet1-SUMO	BamHI, NotI	S
*ct*RpL4	1-341	pETDuet1-SUMO	BamHI, NotI	S
*ct*RpL4	1-328	pETDuet1-SUMO	BamHI, NotI	S
*ct*RpL4	1-311	pETDuet1-SUMO	BamHI, NotI	S

Protein	Residues (Mutations)	Expression Vector	Restriction Sites 5', 3'	N-terminal overhang
*ct*RpL4	1-300	pETDuet1-SUMO	BamHI, NotI	S
*ct*RpL4	1-287	pETDuet1-SUMO	BamHI, NotI	S
*ct*RpL4[a]	1-277	pETDuet1-SUMO	BamHI, NotI	S
*ct*RpL4	1-277	pET28a-SUMO	BamHI, NotI	S
*ct*RpL4	278-365	pET28a-SUMO	BamHI, NotI	S
*ct*RpL4[a]	308-332	pET28a-SUMO	BamHI, NotI	S
*sc*RpL4	1-362	pETDuet1-SUMO	BamHI, NotI	S
*sc*RpL4	1-276	pET28a-SUMO	BamHI, NotI	S
*ct*Kap104	1-938	pGEX-6P-1	BamHI, NotI	GMGS
*ct*Kap-α	81-506	pET28a-SUMO	BamHI, NotI	S
*hs*Ran	1-216 (Q69L)	pET28a	NdeI, BamHI	GPHM
*hs*Kap104[a]	1-890 (337-367 replaced by GGSGGSG)	pGEX-6P-1	BamHI, NotI	GMGS

[a]Constructs that were used for crystallization of Acl4$^{28\text{-}361}$•RpL4$^{1\text{-}277}$ and *hs*Kap104$^{1\text{-}890}$•RpL4$^{308\text{-}332}$

[b]Mutants are listed with both *C. thermophilum* and the corresponding *S. cerevisiae* numbering

SUPPLEMENTARY REFERENCES

1. Sikorski, R.S. & Hieter, P. A system of shuttle vectors and yeast host strains designed for efficient manipulation of DNA in Saccharomyces cerevisiae. *Genetics* **122**, 19-27 (1989).
